# What doesn't kill you makes you poorer: Adult wages and early-life mortality in India

**DOI:** 10.1016/j.ehb.2015.11.006

**Published:** 2016-05

**Authors:** Nicholas Lawson, Dean Spears

**Affiliations:** a Aix-Marseille University (Aix-Marseille School of Economics), CNRS & EHESS, Centre de la Vieille Charité, 2 rue de la Charité, 13002 Marseille, France; b Centre for Development Economics, Delhi School of Economics, University of Delhi, Delhi 110007, India

**Keywords:** Early-life health, Infant mortality, Disease environment, Wages, India

## Abstract

A growing literature indicates that effects of early-life health on adult economic outcomes could be substantial in developing countries, but the magnitude of this effect is debated. We document a robust gradient between the early-life mortality environment to which men in India were locally exposed in their district and year of birth and the wages that they earn as adults. A 1 percentage point reduction in infant mortality (or 10 point reduction in IMR) in an infant's district and year of birth is associated with an approximately 2 percent increase in his subsequent adult wages. Consistent with theories and evidence in the literature, we find that the level of schooling chosen for a child does not mediate this association. Because of its consequences for subsequent wages, early-life health could also have considerable fiscal externalities; if so, public health investments could come at very low net present cost.

## Highlights

• Studies early-life disease environment and adult wages for men in India.
• Robust negative gradient between infant mortality and wages decades later.
• 10 point IMR reduction associated with approximately 2 percent wage increase.
• Not mediated by level of schooling received.
• Due to fiscal externality, public health investments could have low net present cost.

## 1 Introduction

A growing literature documents that workers exposed to better early-life health and less disease in early life have higher human capital as adults. Although this literature has largely focused on developed countries, economists have hypothesized that an effect of early-life health and disease externalities could be importantly larger in developing countries, where disease insults are worse and more varied (Currie and Vogl, 2013, Spears, 2012b). If early-life health indeed importantly limits human capital in developing countries, wages could be a mechanism through which health has important effects on developing economies and, because of income and consumption tax revenue, on the government's budget. However, the magnitude of these effects is a topic of current debate in the development economics literature (Acemoglu and Johnson, 2007, Bleakley, 2010a, Hansen, 2014). It is therefore important to understand and quantify relationships between early-life health and subsequent wages in developing countries (Vogl, 2014).

Our article documents a robust gradient between the health environment to which today's workers in India were exposed as infants in past decades, and the wages which they now earn. We match male workers in nationally representative survey data on wages in 2005 to district-level estimates of infant mortality in their year of birth, which we use as a measure of early-life health and disease, following Acemoglu and Johnson's investigation of mortality and GDP. We then use a double fixed effects (place and time) identification strategy to compare workers in the same district labour market today who were exposed to different mortality regimes when they were born. Our results suggest that being born in a district-year with a higher infant mortality rate (IMR) – and corresponding worse health and disease environment – is associated with a significant but plausible reduction in earnings decades later. A 1 percentage point reduction in infant death (or a 10 point reduction in IMR) in the environment to which an infant was exposed in his year of birth is associated with an approximately 2 percent increase in his subsequent wages as an adult. Because important threats to early life health remain widespread in India and other developing countries, these estimates are of continuing economic and policy importance.

Bleakley (2010a) reviews theory and evidence that disease – and especially child health – could have important effects on adult human capital and income in developing countries. Although substantial effects would be consistent with current theory and recent empirical findings, there is debate about the quantitative importance of disease to economic development. Bleakley further presents a model demonstrating that improved early-life health is likely to increase both the *returns* to schooling and the *opportunity costs* of schooling (in the form of higher earning ability for children and young adults); as a result, improvements in early-life health may not lead to large increases in the optimally-chosen quantity of formal schooling. Our setting allows us to test this prediction, and indeed we find links between improvements in early-life mortality and adult wages, but no association between IMR and subsequent schooling.

Infectious disease is well-known to have negative externalities on neighbors. Because of its consequences for subsequent wages, the disease environment also can have *fiscal* externalities, which are of importance for government cost-benefit analysis in the context of a developing country with limited fiscal capacity and many competing potential expenditures. We apply our estimates to compute considerable consequences for government tax revenues of reductions in income and consumption due to early-life health and disease. We show that relatively modest effects of early-life health on individual economic outcomes can add up to quantitatively important overall economic and fiscal effects, in the context of a developing country with a large burden of disease externalities. One consequence is that public action to improve the disease environment faced by infants could come at very low net present cost to governments (Alderman and Behrman, 2006).

The rest of the article proceeds as follows. Section 2 provides a brief overview of relevant literatures and the Indian context, and Section 3 details our empirical strategy. Section 4 then presents our main empirical results, and Section 5 demonstrates the robustness of our results in several directions and explores the mechanisms driving our findings. Section 6 translates the main empirical results into implied consequences for government revenues and a simple measure of welfare. Section 7 concludes.

## 2 Background

We study adult male workers in a representative survey of India. Children in India today are exposed to a considerable disease burden in early life, which was even greater at the time when today's adults were children. In 1970 – the year before our data begins, when 35 year old workers in 2005 were born – nearly 20 percent of children died before their fifth birthday, and 13 percent of infants died in their first year of life (Unicef, 2012). Infant mortality in India has fallen to about 4.1 percent today, but this still substantially exceeds infant mortality of 1.1 percent in China and 3.3 percent in Bangladesh, a poorer neighboring country. If early-life health and disease is an important constraint on development and income, it would be of considerable importance in India, where about one-fifth of all births occur.

### 2.1 Effects of early-life health on adult economic circumstance

An active literature in economics documents that healthier babies are more likely to become healthier and more productive children and adults (Currie, 2009). Early-life health matters because the first few years are a critical developmental period; children who have better health and net nutrition in early life are more likely to reach their physical growth potentials *and* are more likely to reach their cognitive potentials (Case and Paxson, 2008). Indeed, much of this literature, unable to match adult wages to early-life conditions, has used adult height as a proxy for early-life health (*e.g.* Vogl, 2014). Most of this literature has focused on developed countries (*e.g.* Deaton and Arora, 2009). However, the impact of early-life health in developing countries may be an even more important part of labour market outcomes than in developed countries, relative to heterogeneity in genetic potentials: disease conditions are worse, care and remediation may be less available, and heterogeneity in health insults is likely to be larger than in developed countries.

Economists have linked various measures in the chain from early-life human capital accumulation to its long-run consequences: childhood and adult height, childhood and adult cognitive achievement, and adult wages. For example, Bozzoli et al. (2009) show that people are shorter in countries with higher infant mortality, whereas de Oliveira and Quintana-Domeque (2014) find that GDP per capita in year of birth is the main correlate of height in late-20th-century Brazil; Alderman et al. (2009), Alderman et al. (2006), and Glewwe et al. (2001) show that taller children have greater cognitive achievement; Vogl (2014) shows that taller adults in Mexico earn more money; and many papers in labour and development economics document economic returns to cognitive achievement.

Among the few studies that have been able to directly connect variations in disease environment to gains in wages or consumption are two recent papers by Bleakley (2010b) and Cutler et al. (2010), who estimate effects of early-life exposure to malaria on adult wages in the Americas and on consumption in India, respectively.^2^ Our article expands this literature beyond malaria and links the early-life mortality environment directly to adult wages in a developing country; we then apply our estimates to compute consequences for government revenue, in the context of a large developing economy where our estimates are of continuing relevance.

### 2.2 Endogenous education and the envelope theorem

It is common in the literature linking early-life health to adult wages to verify an intermediate effect on schooling (*e.g.* Vogl, 2014). Bleakley (2010a) presents a standard model in which children's education is optimally chosen to maximize lifetime income. The benefits and costs of education both depend on the child's health, and therefore on the disease environment. Bleakley notes that better health almost certainly increases the returns to schooling, but better health is quite likely to also increase the opportunity cost of schooling in an economy where children can also engage in productive work, as would have been common in India several decades ago. Therefore, it is not clear that the effect of health on education should be positive.

Moreover, Bleakley (2010a) shows that a straightforward implication of the envelope theorem is that the chosen schooling level is unlikely to be an important mechanism of the effect of health on adult earnings: if schooling is chosen to maximize lifetime income, for example, individuals will attain schooling up to the point at which the marginal gain from schooling in terms of added lifetime earnings (accounting for foregone earnings while in school) is zero. Therefore, even if changes in early-life health impact cognitive development in such a way as to increase the quantity of schooling chosen, this change in schooling should have no first-order impact on lifetime earnings; rather, any effect of early-life health on adult earnings should primarily accrue through improvements in human capital independent of the level of schooling.

Because we observe an adult's early-life mortality environment, his wages, and his level of schooling, we are able to test these two theoretical predictions. We find that exposure to better early-life health is indeed associated with earning higher adult wages. However, we find no similar association with education levels, and flexibly controlling for a detailed education vector does not influence the magnitude of the gradient we document between early-life health and adult earnings, as predicted by Bleakley (2010a).

## 3 Empirical strategy and data

In this section, we outline a strategy to quantify the gradient between the early-life mortality environment and adult wages. Although we write about “identification,” we do not interpret our results literally as an effect of early-life mortality *outcomes* on wages; rather, adult economic outcomes and infant mortality are both shaped by an early life health and disease environment, which includes sanitation and other dimensions of public health. We use a double fixed effects (place and time) identification strategy to compare workers who compete with one another within the same labour market today, but were exposed to better or worse disease environments and mortality regimes when they were born.

Our identification strategy exploits two facts about a cross-section of workers of different ages who live near one another:

• First, their wages today are determined, in part, by a common labour market. Insofar as labour is substitutable across workers of different ages, those workers are offering to supply their labour to the same, shared demand side of the market.^3^
• Second, workers of different age cohorts in a cross-section implicitly form a synthetic panel: workers of different ages today represent the effects of early-life health at the different points in history when they were born.

**Fig. 1:**
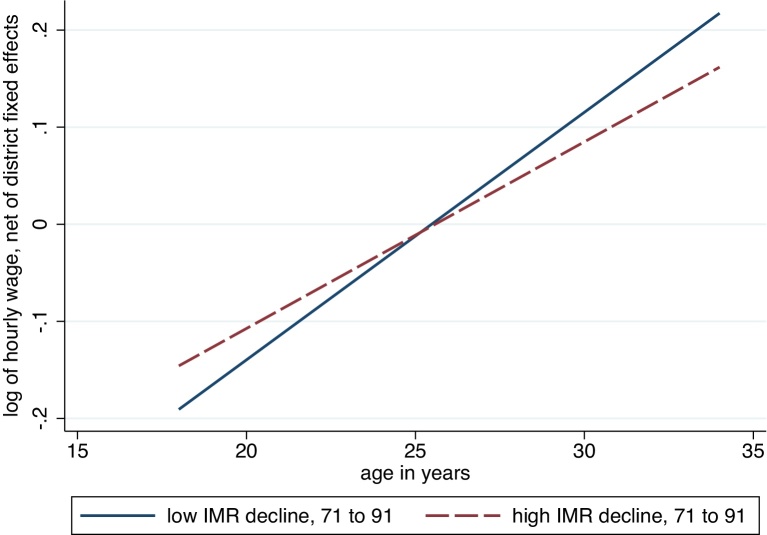
Identification strategy. *Notes*: For the purpose of this figure, our sample of districts has been ordered by the estimated decline in IMR from 1971 to 1991, projected using Census data from 1981, 1991, and 2001; the wage-age relationship in 2005 for the half of districts with the smaller decline is presented with the solid blue line, while that for the half of districts with the larger decline is depicted using the dashed red line. (For interpretation of the references to color in this figure legend, the reader is referred to the web version of the article.)

We exploit district-by-time variation to investigate the association between adult wages in 2005 and early-life health in districts throughout India in the 1970s and 1980s. In particular, we match historical, district-level census data on early-life mortality with cross-sectional survey data on adult wages. Fig. 1 presents our identification strategy graphically. The graph plots average wages, net of district fixed effects, as a linear function of age. The most visible feature of the graph is the upward slope: within essentially all Indian districts, older workers are paid more than younger workers, on average.

Our identification is found in the *difference* between the two slopes. Early-life health improved over time in essentially all districts. However, these improvements were not uniform across districts. In districts where the mortality environment improved more quickly over this period, younger workers would be expected to have relatively better early-life human capital than older workers in the same district, in comparison with the difference between older and younger workers in other districts where the health environment improved more slowly. Therefore, our identification strategy asks whether the positive gradient between ages and wages is *less steep* in districts where the mortality environment improved *more quickly*. An initial answer is visible in the difference in the two slopes in Fig. 1: the age profile of wages is less steep in the half of districts with above-average declines in infant mortality.

### 3.1 Sources of historical and contemporary data

We match data from two different sources. For our present-day dependent variables and control variables, we use data on individual adult males from the India Human Development Survey (IHDS), a nationally representative 2005 cross-sectional survey of 40,000 households (Desai et al., 2007). We study men born between 1971 and 1989, who were therefore between 16 and 34 years of age in 2005, leaving us with 12,783 observations, as can be seen in Table 1. These observations are drawn from 277 districts across 17 states, including the 13 most-populated states as of the 2011 Census which comprise over 85% of the total population; the full list of states covered by our data can be found in Online Appendix B.2.^4^

Our primary dependent variable is the log of hourly wages in rupees, as computed by the IHDS.^5^ As a robustness check and an input to our welfare computations, we also estimate the gradient between the early-life mortality environment and household consumption per capita.

For our independent variable, we use historical infant mortality rates, a standard variable in the economic history and economic demography literatures. Infant mortality rates (IMR) are scaled as the number of deaths in the first year of life per 1,000 live births. Economic historians have long used infant mortality as a measure of the disease environment. Because of consequences of disease for net nutrition, early-life infant mortality rates are increasingly well understood to be an important determinant of height in developing countries today (Bozzoli et al., 2009), and historically in now-rich European countries (Hatton, 2014).^6^

Of course, we do not literally estimate *effects* of infant mortality: we would not expect average wages in a district to rise as a result of an emergency medical intervention that barely prevented the deaths of the marginally last infants to die. Instead, we interpret the gradient that we document between mortality and subsequent wages to reflect the influences of the health and disease environment on both. In the 1970s and 1980s, infant mortality would have been importantly shaped by infectious disease and maternal nutrition, rather than by perinatal medical care; the first recorded data on the percent of births attended by any skilled medical staff in the World Bank World Development Indicators is 34.2 percent in 1993.

We match district-level historical IMR data from various rounds of the Census of India to the IHDS. Census data is available only at 10 year intervals, specifically in 1981, 1991 and 2001; such ten-year intercensal periods are standard in demographic data, and infant mortality data at the district level does not exist for any Census prior to 1981. We use these three Census rounds to estimate a long difference in IMR by district: we run a district-specific linear regression of census IMR on year for each district, and use regression coefficients to predict IMR for each year from 1971 to 1989, thus matching the adults we study with the predicted IMR in their district, in the year of their birth. Results are robust to the alternative use of log-linear regressions, or instrumenting for the linearly-predicted IMR with the log-linear prediction to deal with potential measurement error in infant mortality, as we will show.^7^ Given the 10-year interval between each Census round, we do not project IMR estimates any further into the past than 10 years prior to the 1981 Census, which determines the start date of our data as 1971. This procedure provides the best possible estimates of infant mortality rates by district over the 1971–89 period, but our estimates are not sensitive to the start date; a robustness check in Online Appendix D shows that our point estimate is numerically very similar – and even slightly larger in absolute value – if we omit all years prior to 1981.

In a subsequent robustness and plausibility check, we use district-level sanitation rates, operationalized as the percent of households in a district who own a toilet or latrine, rather than defecating in the open. Early-life exposure to open defecation in India has recently been shown to be a significant predictor of infant mortality, childhood height (Spears, 2013) and childhood cognitive achievement (Spears and Lamba, 2016).^8^ We also take this historical data from the Indian Census from 1981, 1991 and 2001, where in this case data is available separately for the urban and rural portions of each district (when a district contains both). Rural open defecation is not observed in the 1981 Census, but the World Health Organization estimated that as of 1980, only 1% of India's rural population had access to any sanitation facilities, so we assign 100% rural open defecation in 1981,^9^ and predict sanitation rates for each observation by performing separate district-specific regressions for rural and urban sections of each district. As a result, we use a smaller, younger sample in the sanitation robustness check; rural open defecation was almost universal before 1981, and therefore there is no improvement to be studied.

Table 1 presents summary statistics for the IHDS and census data that we use. Our average workers are poor – earning about 10 rupees an hour, or about $0.23 in 2004 dollars (roughly $1 at purchasing price parity) – and were exposed to threatening early-life health environments, with infant mortality of 113 deaths per 1000 births and only about 17 percent sanitation coverage, on average. The maximum estimated IMR in our sample is 281, and the minimum is 29.7; the average individual lives in a district in which the IMR declined by 2.8 points per year, but there is considerable variation in this measure, ranging from a 10 point reduction per year to a 1.8 point increase. The workers we study are also relatively young, with an average age of about 26 years.

### 3.2 Empirical specification

Our identification strategy asks whether the present-day age profile of wages is less steep in districts where early-life health has improved more quickly. In such districts, younger workers will have had better early-life human capital accumulation compared to older workers, relative to the difference between younger and older workers in other districts with slower improvements in health. To quantify this health gradient of wages, we estimate the following district-by-year double fixed effects regression:

(1) $$\ln(y_{\mathrm{idt}})=\beta {\mathrm{IMR}}_{\mathrm{dt}}+X_{\mathrm{idt}}\theta +\alpha_{d}+\gamma_{t}+{ɛ}_{\mathrm{idt}}$$

where *i* indexes individual adult workers, *d* denotes districts, *t* represents years of birth, and *X* is a set of control variables. We use district fixed effects *α* to control for any average differences in labour markets across districts or any difference in the initial level of early-life health, and year fixed effects *γ* to account for the overall age profile of wages. Standard errors are clustered conservatively at the district level (277 districts are represented in our data), clustering all individuals in a district regardless of their year of birth or within-district primary sampling unit (Cameron and Miller, 2015).

It is important to note that any coefficient on *IMR*_*dt*_ that we observe can only be consistent with a factor that changed over time within districts in parallel with improvements in early-life health, and which, in a contemporary cross-section, differentially impacts people who were born a few years apart; that is, people who would have been subjected in similar ways to changes in village infrastructure, education, or cultural norms. Because these wages are observed in a cross-section of workers of different ages, any difference across local labor markets would be absorbed by district fixed effects. Our specification thus rules out many forms of spurious correlation driven by factors other than early-life health. To further demonstrate the robustness of our strategy, as well as the stability of our coefficient estimate, we add controls *X*_*idt*_ in stages:

• state-specific linear time trends: identifies the effect of early-life health from the extent to which the district time trend in IMR differs from the state-wide time trend, to rule out any spurious state-level omitted variables;
• state × urban fixed effects: controls for a separate rural–urban difference in each state;
• state × social group indicators: state-specific indicators for eight caste and religion groups;
• female literacy: district-level female literacy in the district and year of the man's birth, matched from census data in the same way as IMR.

The control for female literacy – another indicator of human development and an important determinant of early-life human capital – helps ensure that we are identifying off of variation in early-life health and the disease environment rather than improvements in other district facilities and outcomes, and also verifies that no spurious correlation is mechanically introduced by our district-level matching process.

We will additionally add a further set of covariates which may entail *overcontrolling*, relative to a properly specified model, but which will allow us to further rule out omitted variable bias while investigating possible mechanisms of the effect we document. In particular, we will add indicators for the worker's membership in seven job categories,^10^ which rules out spurious structural differences in district labor markets.

Finally, we will add detailed indicators for years-of-school interacted with literacy. These additions would be overcontrolling if education investments were partially caused by early-life health. However, as we have discussed, models of optimal investment in education suggest that improvements in early-life health could increase adult wages without having a large effect on schooling decisions. Moreover, schooling may not be an important mechanism of the translation of early-life health into adult wages. In a test of this theoretical prediction, we will show that our empirical strategy finds no effect of early-life IMR on education, and no impact on our main coefficient of interest when we include education variables in the regression.

Our strategy implicitly assumes that the young adult men in our sample were born in the same district in which they lived at the time of our data. This is a reasonable assumption, because permanent migration for adult *males* in India is relatively uncommon (Rosenzweig and Stark, 1989); in contrast, women often migrate at the time of marriage to join their husbands’ households.^11^ We will demonstrate that our results are not affected by migration: permanent migration is observed in the IHDS, and excluding the small fraction of men who have ever moved residences does not meaningfully change our coefficient estimates.

## 4 Empirical results

**Fig. 2:**
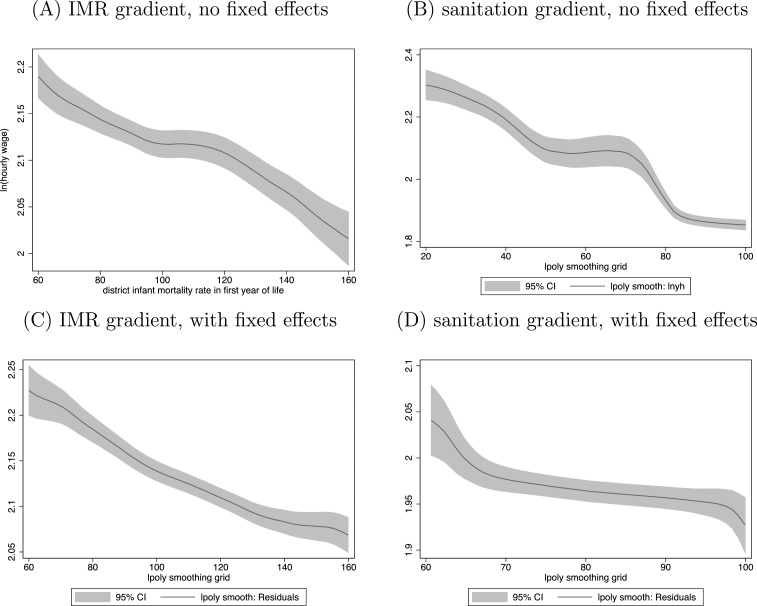
Worse early-life disease environment associated with lower adult wages. *Notes*: All panels present weighted kernel regressions of wages on district-level IMR or open defecation. In panels C and D, the regressions include year-of-birth and state-times-urban fixed effects as controls. Bandwidths are 15 in panels A and C, and 8 in panels B and D.

Are men who were exposed to a better early-life health environment, as measured by infant mortality, subsequently paid higher wages as adults? As an initial answer to motivate our main result, Fig. 2 verifies that men who were born in district-years with worse infant mortality and sanitation earned lower wages as adults in the IHDS in 2005. Panels A and B plot locally weighted kernel regressions depicting a clear downward trend. Panels C and D plot residuals of wages against residuals of our health measures, in both cases after controlling for year-of-birth and state-times-urban fixed effects, with the means added back in to make the range of the figures comparable to A and B. The visible downward trend remains, in an initial suggestion of the gradient we will estimate.

### 4.1 Main result: adult wages and early-life mortality rates

Table 2 presents our main empirical result: men who were born in district-years with higher infant mortality have lower adult wages, on average. The regression coefficients imply that a 1 percentage point increase in the infant mortality rate (that is, 10 more infant deaths per 1000 live births) would be associated with a decline in adult wages of almost 2 percent.^12^ The mortality-wage gradient is notably stable across regression specifications. In particular, similar results are found if IMR is projected linearly across survey rounds, as in Panel A, or if linear prediction is instrumented for with log-linear projection, as in Panel B, to reduce measurement error.

Adding a long vector of regression controls fails to importantly change the coefficient estimate. In particular, column 2 controls for state-specific linear year-of-birth trends (age gradients), separate urban indicators for each state, and separate religious and caste indicators for each state; the coefficient remains essentially identical, and if anything slightly increases.

Column 3 goes further by including indicators for job categories, which may well be in part a *consequence* of early-life health and human capital accumulation (Vogl, 2014). This does not change the coefficient on early-life IMR exposure. This suggests that job categories are not omitted variables that are spuriously responsible for our main result and that early-life health does not appear to be related to wages through the mechanism of sorting into these categories.^13^

Column 4 tests Bleakley's envelope theorem observation: chosen schooling should not mediate the relationship between early-life health and adult wages. Controlling flexibly for education, measured as a vector of school-grade indicators interacted with literacy, has no effect on the coefficient on birth-year infant mortality. Rather, the link operates through improvements in human capital at the same level of schooling. Note that this is not merely because the schooling variables are too noisily measured to have a signal; in the regression of column 4 of panel A, the vector of education indicators is highly statistically significant with a test statistic of *F*_31,276_ = 19.69, *p* < 0.00001.

Finally, column 5 controls for census female literacy rates in the district and year of the man's birth; although the sample decreases slightly because this cannot be matched to all district-year combinations, the stability of the coefficient suggests that the gradient we observe is due to the early-life *health* environment, rather than historical human development more broadly.^14^

A robustness check in Online Appendix D further demonstrates that our coefficient estimate is stable across different start dates for our data; in fact, the association between IMR and subsequent wages even becomes a bit *stronger* when data on individuals born in the 1970s is discarded.

### 4.2 Bleakley's optimization result: no effect on education

Improved early-life health could increase adult human capital by increasing physical strength or cognitive ability directly, or by increasing attained schooling, which would in turn increase wages. However, Bleakley (2010a) predicts that the optimally chosen quantity of schooling should not be an important mechanism linking early-life health to adult wages, and that schooling may not even increase in response to an increase in early-life health, because health increases both the benefits and costs of a child attending school. We have seen evidence for the first claim; in Table 3 we test the second. Neither the early-life environment proxied by IMR nor the level of sanitation (which we will consider as a robustness and mechanism check in subsequent sections) are associated with a difference in schooling levels. To emphasize, this is not because the schooling variables are unreliable noise: they are quite significant predictors of adult wages.

## 5 Robustness and mechanisms

In this section, we present three further empirical tests of the robustness of our main result and of the plausibility of a link between the early-life health environment and adult wages. First, we show that early-life exposure to open defecation – one important determinant of infant mortality in India – is similarly associated with adult wages. Then, returning to the concern that we observe only a man's district of residence and not of birth, we rule out migration as a cause of our result by showing that the result is similar when migrants are excluded. Finally, we document a similar association between the early-life health environment and household consumption, but only for households in which the man we study is the main earner. This will be an input into our welfare calculations, and is an important plausibility check for the consistency of our result with economic mechanisms, rather than it reflecting a spurious correlation.

### 5.1 Early-life exposure to open defecation

Among potential health insults, exposure to poor sanitation is particularly likely to have quantitatively important consequences for adult economic outcomes because its effects on early-life health are so large. Water and sanitation are known to be important determinants of health outcomes, especially infant mortality (for recent examples in economics, see Cutler and Miller, 2005; Galiani et al., 2005; and Watson, 2006). Nonetheless, the associations between human capital and sanitation and disease have received relatively less research attention than human capital gradients in income and education. However, a recently active literature has shown that sanitation can be particularly important in human capital accumulation in developing countries, especially in India, where open defecation – without using a toilet or latrine – is particularly widespread (Spears, 2013).

Open defecation matters for health because it releases fecal germs into the environment which cause disease in growing children. According to Unicef and WHO (2012) statistics, over a billion people worldwide defecate in the open; most of these live in India, and most people who live in India defecate in the open. As a large medical and epidemiological literature documents, ingestion of fecal pathogens as a result of living near poor sanitation is well-known to cause diarrhea (Esrey et al., 1992). Checkley et al. (2008) use detailed, high-frequency longitudinal data from five countries to demonstrate effects of childhood diarrhea on subsequent height. In addition to the obvious threat of diarrheal disease, open defecation can cause net nutritional insults through worm or other parasitic infections, or by increased energy consumption fighting disease. Most recently documented in detail in the medical literature, but perhaps very important, is the possibility of widespread chronic but subclinical environmental enteric dysfunction (Humphrey, 2009). Economists have further identified effects of childhood exposure to sanitation-related disease on human capital (Bleakley, 2007, Baird et al., 2011).

Substituting sanitation coverage for infant mortality as the independent variable in our regressions allows us to provide further evidence of a link between the early-life disease environment and adult wages. Open defecation is only one of many important causes of historical and contemporary infant mortality in India, and as such sanitation and IMR are far from perfectly negatively correlated: among individuals in our baseline wage regression for whom both IMR and sanitation data are available, the correlation is −0.196. In fact, the correlation between the changes in IMR and sanitation that we use for identification is essentially zero: in fact, it is slightly positive at 0.0637, because districts that saw the greatest reductions in IMR tended to be the districts with the worst disease environment, and thus with the highest starting IMR, whereas sanitation in those districts tended to improve more slowly.^15^ Therefore, the regressions below make use of a second, and quite different, source of variation in early-life health environment, making it less likely that both are simply capturing some other unobserved variable. Thus, insofar as results using sanitation are broadly similar to results found using IMR, we interpret this concordance as indicative that the mortality results are plausibly consequences of the disease and health environment.

Table 4 reports regression results with sanitation coverage as the independent variable. The sample is smaller in Table 4 than in the IMR analysis because we only use data on individuals born in 1981 and after, as rural open defecation was almost universal before this period, meaning there was no improvement to study; this smaller sample will decrease the precision of coefficient estimates.

As the table shows, we indeed find a gradient of important but plausible magnitude between early-life sanitation and adult wages. The main independent variable is the percent of households owning a toilet or latrine, rather than defecating in the open. A 10 percentage point decrease in open defecation translates into an approximately 2–3 percent increase in wages. Thus, we find that in districts where sanitation has improved more quickly over time, the within-district wage profile is less steeply increasing in age. As before, our result is quantitatively stable as a long vector of controls is added, including for state-specific time trends, caste and religious groups, and state-specific urban residence. Also as before, adding job categories and education indicators – although these are, themselves, predictive of wages – does not change the coefficient on early-life sanitation.^16^

Are these coefficient estimates of plausible size? Although no comparable estimates exist in the literature, we can make an approximate guess of a plausible magnitude of the gradient we estimate by multiplying quantities that do exist in the literature:

(2) $$\frac{\%\Delta y}{\Delta \mathrm{open}\;\mathrm{defecation}}=\frac{\Delta \mathrm{height}\;(\mathrm{sd})}{\Delta \mathrm{open}\;\mathrm{defecation}}\times \frac{\Delta \mathrm{height}\;(\mathrm{cm})}{\Delta \mathrm{height}\;(\mathrm{sd})}\times \frac{\%\Delta y}{\Delta \mathrm{height}(\mathrm{cm})},$$

where *height (cm)* is adult height in centimeters and *height (sd)* is child height-for-age in standard deviations, and where %Δ*y* is the percentage change in wages. We assume that a one standard deviation increase in child height becomes a one standard deviation increase in adult height, and substitute 6.9 cm for $\frac{\Delta \mathrm{height}(\mathrm{cm})}{\Delta \mathrm{height}(\mathrm{sd})}$, the standard deviation of Indian adult male height in the most recent Demographic and Health Survey. We combine three estimates of $\frac{\%\Delta y}{\Delta \mathrm{height}(\mathrm{cm})}$ from the literature: Vogl's (2014) from Mexico, and high and low estimates for men in the U.S. from Case and Paxson (2008). We use five estimates of $\frac{\Delta \mathrm{height}(\mathrm{sd})}{\Delta \mathrm{open}\;\mathrm{defecation}}$: Lin et al. (2013) from Bangladesh, Kov et al. (2013) from Cambodia, and one estimate from Spears (2012a) and two from Spears (2013) from India.^17^

Crossing these produces 15 predictions of the coefficient for wages regressed on early-life sanitation coverage. These range from 0.0006 to 0.0052, with a median of 0.0017 and a 75th percentile of 0.0021. This is exactly the neighborhood of our estimates in Table 4: 0.0018–0.0032. Although our estimates are slightly above the median of these estimates, there is reason to suspect our estimate would be larger: a higher fraction of the variation in height in India reflects early-life health than in the U.S. or likely even than in Mexico. Indeed, Spears (2012b) finds that the gradient between height and cognitive achievement is much steeper for Indian children than for U.S. children. Therefore, we conclude that our estimates are quantitatively consistent with predictions from estimates in the literature.

### 5.2 Results are not driven by migration

Because we observe the district in which men live, not the district in which they were born, certain patterns of endogenous migration could, in principle, bias our estimates.^18^ Fortunately, the IHDS includes data on migration, so we can assess the importance of this concern. The data report whether a particular individual moved homes since their birth, though we cannot see where they migrated from, so we are likely to overstate migration as many moves would be within the same district. We run the regressions of wages on IMR and sanitation only for stayers, by omitting all individuals who report ever having moved (even those who move within districts). The results are presented in Table 5, and the coefficients are nearly unchanged. Further analysis in Online Appendix C finds no evidence that selective migration along the dimension of improvements in early-life health occurred in any case. Therefore, selective migration does not appear to be responsible for our results.

### 5.3 Effects on consumption

In this section, we extend our analysis to check for a gradient between early-life mortality and household consumption per capita. In part, this is a robustness check of our main results: we would expect an increase in income to increase household consumption, especially if the adult male we study is an important source of household income. Additionally, these estimates will be used in our fiscal and welfare computations: consumption taxes – such as value added tax – are a larger fraction of government revenue in India than income tax, so if we are concerned about the fiscal impacts of the early-life health environment, it is important to confirm that consumption is affected as well.

Table 6 documents the association between early-life IMR and the log of household monthly consumption per capita.^19^ Column 1 repeats the estimate of the gradient between early-life IMR and *wages* from Table 2. Column 2 shows that the association with household *consumption* is of similar magnitude, although slightly smaller. Importantly, however, the adult men whom we are able to study are relatively young, and only some of them will be significant earners for their households. One common family structure in India is a joint household where adult men and their spouses live with the man's parents; such households could have multiple brothers and a father earning income. Column 3 restricts the sample to the approximately two-thirds of the men we study who earn the most money of all people in their household; the effect is quantitatively similar to the effect on wages in column 1.

Column 4 presents results for men who are not main earners; their early-life health environment has no detectable effect on their households’ consumption. This non-finding is important because it is consistent with what the economic demography of the Indian context would predict; this therefore suggests that our finding is not merely a spurious reflection of correlation between some aspect of households’ socioeconomic status today and the health environment in their districts in past decades.^20^ For example, our results are not driven by factors that impact the entire family: if IMR or sanitation in birth year was simply capturing general improvements in village infrastructure, the impact should be felt by all earners today, not just those families where the current primary earner was an infant at the time of the improvement.

## 6 Fiscal and welfare implications

Our analysis so far has produced coherent evidence that improvements in the mortality environment are associated with higher subsequent wages and consumption. As a result, we expect that such improvements would have positive consequences for the tax revenues collected by the Indian government. Importantly, this suggests that investments in improved early-life health, such as investments that lead to increased use of improved sanitation, could come at a low net fiscal cost to the government of a country such as India.

It is also likely that higher income and consumption will translate into increased welfare for Indian households. However, this is not certain in a context of non-unitary households; Indian households are often large and complex, and it is beyond the scope of our analysis to evaluate who receives the increase in consumption within a household, and how that might affect intra-family relations or bargaining power. The IHDS does not observe person-level consumption, only household-level. Thus, we can evaluate the impact of improvements in the early-life disease environment on household consumption, and aggregate the gains up to an economy-wide level, and for simplicity we will refer to these as welfare gains; but it should be understood that we do not claim that increases in household consumption can be monotonically translated into gains in actual person-level welfare. Our estimated consumption gains could more accurately be interpreted as increases in *potential* household well-being, while the actual welfare gains could, in principle, be larger or smaller than what we estimate.

Therefore, in this section, we translate the empirical estimates from the previous sections into fiscal and welfare terms, to provide an illustration of the aggregate impact of early-life health on the Indian economy. For example, if a 1% point reduction in IMR is associated with 1.74% higher wages, we use details of the Indian tax system to estimate the associated increase in future tax revenues, as well as the increase in after-tax income and thus consumption. In each case that we study, the gains in tax revenue and consumption at an aggregate level are large, at least $10 billion in present-value terms; these results demonstrate that improvements in early-life health are associated with substantial gains to the government and to the Indian population. Independent of the accuracy of our main empirical estimates, this section is important in the context of Acemoglu and Johnson (2007), Bleakley (2010a), and others for computing what moderate microeconomic relationships of the sort that we estimate *could* add up to in a large economy.

### 6.1 Fiscal externalities of early-life health

We begin with an analysis of the association between early-life mortality environment and tax revenues, using the result from column 1 of Table 2 as our baseline estimate: a 1% point increase in IMR is associated with 1.74% lower wages. We then assume that the impact of IMR on tax revenues is also 1.74%; thus we conservatively assume that the income elasticity of tax revenue is 1, even though studies of both developed and developing countries tend to find that increases in income lead to proportionately greater increases in tax revenues.^21^

We limit our attention to the income tax and excise and service taxes, as these are taxes which depend directly or in a close indirect way on income and consumption; we ignore customs duties as well as the corporate tax, even though one might expect more productive workers to lead to larger corporate profits. The revenue from these taxes amounted to about 5.11 trillion rupees in 2012–13, or $93.64 billion US;^22^ we assume that a normal working life is 40 years, from 18 to 57, and assume that each year-of-birth cohort produces an equal share of the tax revenue, or $2.34 billion per cohort, prior the change in IMR being considered.^23^

Because these revenue gains occur in the future, starting when the year-of-birth cohort born today enters the labour market, and gradually phasing in after that as more “treated” cohorts enter, we can use a 3.81% discount rate^24^ to add up these future gains and express the revenue gains as a present-value equivalent. This simple procedure allows us to calculate the expected effect on tax revenues from a 1% point reduction in IMR starting today and for each of the next 100 years.

**Fig. 3:**
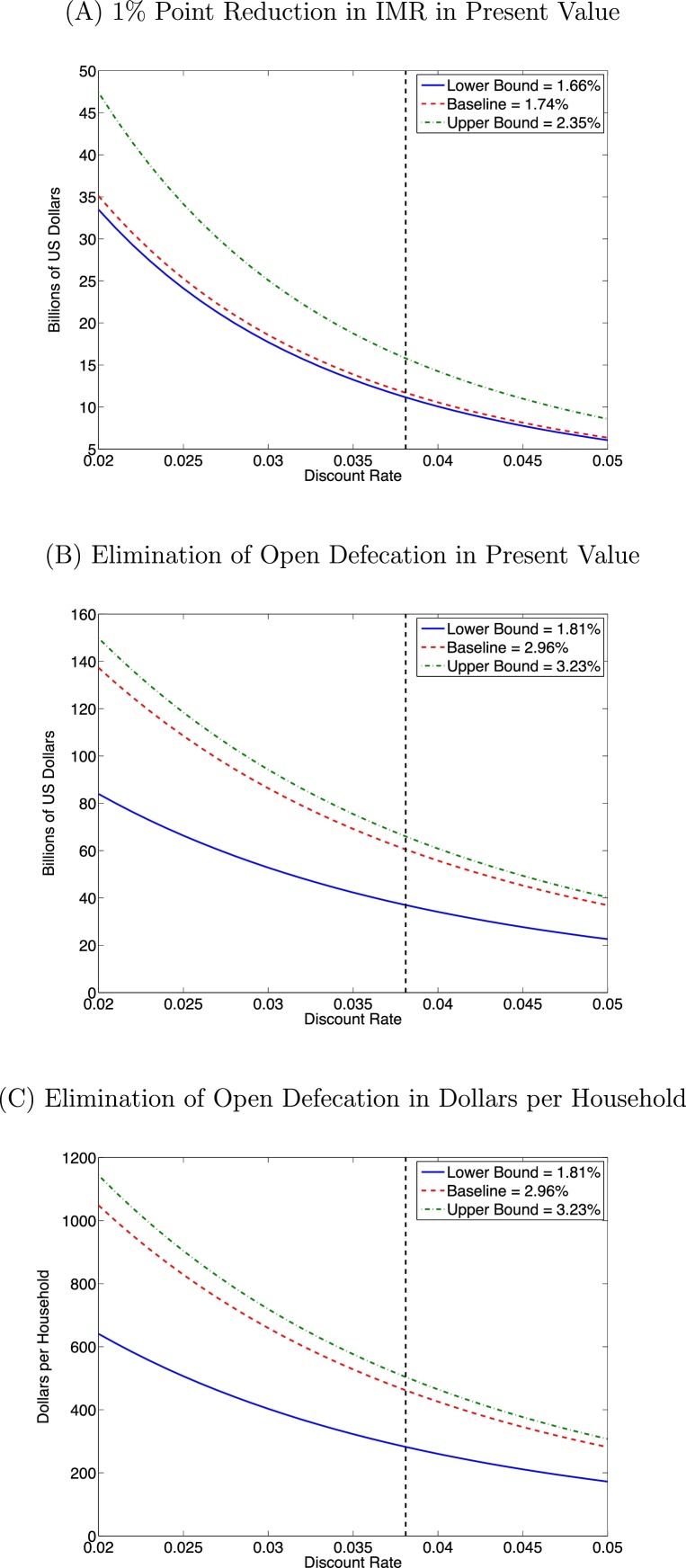
Increases in tax revenues. *Notes*: All panels present present-value tax revenue gains as a function of the discount rate, for three different estimates in each case. Panel A presents the revenue gain from a 1% point reduction in IMR, while panels B and C present revenue gains from the elimination of open defecation, with the latter expressed per household induced to stop defecating in the open. The black vertical dashed line is at the baseline discount rate of 3.81%.

Reducing IMR by 1% point produces fiscal gains starting 18 years from now when the first treated cohort enters the labour market, and ending 157 years from now when the final cohort exits the labour market; the details of the calculations are relegated to Online Appendix E.1, but simply adding up the revenue gains from each cohort and discounting, we find that the sum of the revenue increases is equivalent to $11.70 billion in present value terms.^25^ As a sensitivity analysis, we have also evaluated the revenue gains for the highest and lowest estimates in panel A of Table 2, and for a range of values for the discount rate; the results are displayed in panel A of Fig. 3. The revenue gain depends on both the association between IMR and wages and the discount rate, but especially on the latter, ranging from about $6–9 billion with a rate of 5% to as much as $47 billion with a 2% discount rate. The black dotted line in the figure shows the baseline discount rate of 3.81%.

As a further illustration of the potential fiscal gains from investments in improving early-life health, we also consider the gain in tax revenues associated with a more specific public health objective: the elimination of open defecation today, using our estimate of the association between sanitation and adult wages in Table 4. It was estimated that 53.1% of Indian households defecated in the open in 2011, but that number had been declining at an average rate of 1.05 percentage points per year over the previous decade. Therefore, when considering the elimination of open defecation, the appropriate counterfactual is one in which open defecation continues to decline over time; we assume a continued decline at the same linear rate, so that absent any intervention open defecation would be eliminated in about 50 years. Then, using the result from column 1 of Table 4 as our baseline estimate, where a 1% point increase in sanitation coverage is associated with 0.296% higher wages, we perform a calculation similar to the one above and find a total present-value revenue gain of $60.48 billion; details can again be found in Online Appendix E.1.

The purpose of these calculations is to illustrate the potential quantitative economic importance of early-life health. If we take our results literally as capturing the causal effect of sanitation coverage on wages, then this implies that if there existed an investment capable of eliminating open defecation today at a cost of $60.48 billion or less, there would be no net cost to the Indian government, as those expenditures would be made up in future tax revenues, even after those future gains were discounted at a 3.81% rate. To provide an estimate of the revenue gain per unit of investment (households induced to use latrines), we divide this total by the number of households currently estimated to defecate in the open, which is approximately 131 million, and find that the revenue increase is $462 per household that is induced to stop defecating in the open. In panels B and C of Fig. 3, the range of values generated by trying different discount rates and using the upper and lower bounds from Table 4 are displayed; the gains per household are around $200 at the low end and over $1100 at the top end.

These revenue gains associated with improvements in the early-life public health environment are substantial, and on top of the numerous conservative assumptions made earlier, we have ignored other potential sources of fiscal gains, such as reduced public health care expenditures and calorie requirements if sanitation investments lead to improvements in health among the affected population. Additionally, in Online Appendix F we present the results of quantile regressions, which show that the association between the early-life health environment and wages tends to be larger towards the upper end of the income distribution; as a result, in Online Appendix G.1, we show that the estimated fiscal benefits using the quantile regression results are even larger than those presented here.

### 6.2 Consequences for household economic well-being

Increases in wages associated with improved early-life health not only raise tax revenues; they should also lead to higher after-tax income and consumption. In this subsection, we attempt to quantify these gains in household economic well-being, where, as stated at the beginning of this section, we loosely interpret increases in household consumption as gains in welfare. As throughout the article, we abstract from all benefits of better health and more physically and mentally capable citizens, as we are unable to measure them, and focus only on the gains from higher consumption.

We consider the increase in consumption associated with the same changes as in the fiscal calculations: a 1% reduction in IMR today and for the next 100 years, and the elimination of open defecation. Per-capita GDP was estimated to be $1219 in 2010–11, and given that the 2011 Indian Census finds that about 40% of the overall population are workers,^26^ this implies an average income of $3063 for employed individuals. Tax revenue was estimated to be 10.39% of GDP in 2011, according to the World Bank, so we use a net-of-tax rate of 0.8961, implying after-tax income of $2745 for the average employed individual, and we continue to use 3.81% as the annual discount rate, although now this should be understood as either a personal or social rate of time preference.

**Fig. 4:**
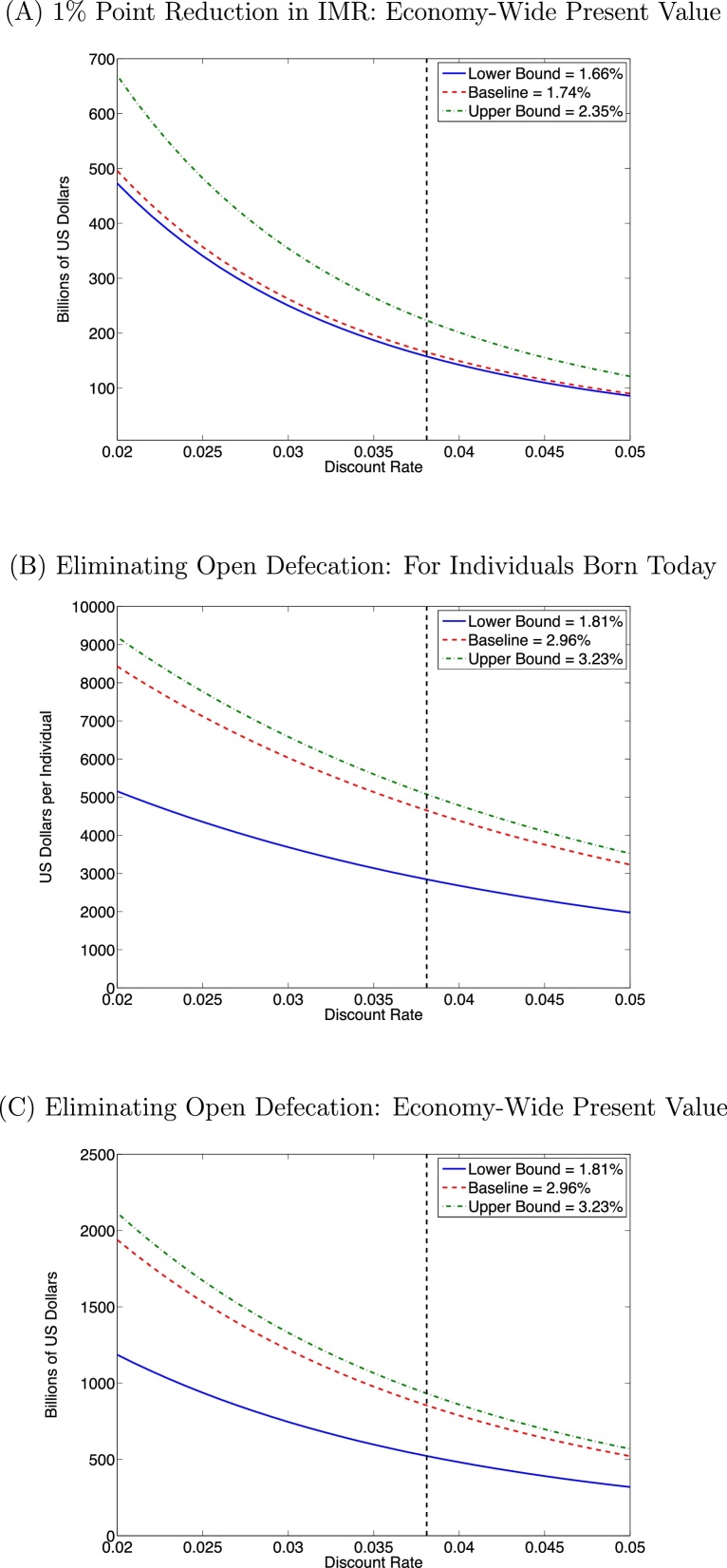
Increases in welfare. *Notes*: All panels present present-value welfare gains from consumption increases as a function of the discount rate, for three different estimates in each case. Panel A presents the economy-wide gain from a 1% point reduction in IMR, while panels B and C present welfare gains from the elimination of open defecation, with B in per-individual terms for people born today, and C the economy-wide gain. The black vertical dashed line is at the baseline discount rate of 3.81%.

In our baseline estimates, each 1% point reduction in IMR is associated with an increase in wages of 1.74%, implying a $47.76 per year increase in household consumption per average worker. The details of the calculations can be found in Online Appendix E.2, and the present-value increase in welfare is equivalent to $165.15 billion of consumption; to put this number in context, it is equivalent in welfare terms to a $6.06 billion (about 0.3% of GDP) increase in annual consumption now and for every year in the future. Panel A of Fig. 4 displays the robustness of this result to varying estimates and discount rates, confirming a significant welfare gain that ranges from about $86 billion to as much as $669 billion.

As an alternative robustness check, we can also use our estimates of the association between IMR and consumption directly. Average per capita consumption was 1430 and 2630 INR per month in rural and urban areas in the 2011 NSS; since 68.84% of India is rural, this implies average consumption of 1803.92 INR per month, or $458.41 per person per year. Each 1% point decrease in infant mortality is associated with an increase in average consumption by 1.73% for everyone in a household with an affected main earner, or $7.93 per person per year. Adding up these gains as described in Online Appendix E.2, we find a total welfare gain of $68.91 billion in present value terms associated with 10 fewer infant deaths per 1000 births. This value is smaller than the one calculated from the wage regressions, which should not be surprising as the estimates of per capita consumption in the NSS are considerably smaller than the after-tax value of per-capita GDP; however, a gain of this magnitude is still economically very significant.

Meanwhile, a 1% point reduction in open defecation is associated with a 0.296% increase in wages, which translates into a $9.07 per year increase in family consumption for an average worker. This implies that the elimination of open defecation would be associated with total discounted gains of $4653 for each worker born today; this is equivalent to nearly four times the current GDP per capita, or about 71.7 years of maximal annual earnings from NREGA, a large government workfare program. Over the entire workforce, the total present-value after-tax income gains over the next 100 years or so are $853.99 billion; as before, this can be expressed as a yearly increase in consumption now and every year in the future, and in those terms it amounts to $31.3 billion per year, or about a 1.7% increase in the current GDP of India. Panels B and C of Fig. 4 displays the robustness of these results; the gains are large at all combinations of parameters, and reach as high as about $2.1 trillion in total, or $9197 per individual born today. And to emphasize again, all of these estimates of “utility” impacts refer only to utility from increased consumption, and not from any other benefits of improved health, cognitive achievement, or mortality; of course, we also abstract from the complications of calculating welfare in a non-unitary household.

Finally, Online Appendix G.2 evaluates the welfare gains associated with improvements in IMR and sanitation using the results of the quantile regressions from Online Appendix F. The quantile regressions suggest that the effect is stronger at high incomes, and if this is true then the welfare gains should be smaller than in the baseline analysis if marginal utility diminishes as income increases. Accordingly, using log utility, we find that the welfare gains are all smaller than those discussed above, but always highly economically significant, with present-value gains of $33–$74 billion from a 1% point IMR reduction and $412 billion from the elimination of open defecation.

Whichever set of estimates or procedure for calculating welfare is used, the estimated gains associated with improvements in early-life health are potentially very large.

## 7 Conclusion

This article documents a robust gradient between the early-life health environment and adult wages, decades later. Exploiting heterogeneity across Indian districts in the time-paths of improvement in infant mortality, we find that men exposed to a better early-life health environment earned significantly but plausibly higher wages as adults. The estimated gains are similar across a wide range of specifications and sets of fixed effects; are replicated in an analysis of historical changes in sanitation; and are consistent with finding improvements in consumption of similar magnitude, precisely when the man for whom we have data is the household's main earner. These results are not spurious consequences of selective migration, which we can observe in our data. Our results are not driven by changes in education; this is consistent with Bleakley's Envelope Theorem prediction and with evidence from Cutler et al. (2010) on the effects of early-life malaria exposure on subsequent consumption in India. Moreover, the apparent unimportance of education to the health-wages relationship may make certain omitted variable threats less likely, such as coincidental other improvements in education or other human capital facilities in the same districts. These findings suggest that early-life exposure to infectious disease could have appreciable consequences for economic outcomes in developing countries, especially in contexts such as India's, where relatively high early-life mortality rates and exposure to high levels of open defecation both continue today.

Relatively modest effects of early-life health on wages – of the magnitude of the gradients that we estimate – could add up to important fiscal consequences. Because improving health raises wages and consumption when children become adults, reductions in infant disease today causes positive fiscal externalities in the future. Wage gains occur decades after improvements in the early-life health environment, so the present-day benefit depends on the interest rate. Our results indicate that public investments to improve the early-life health and disease environment – potentially including efforts to reduce exposure to open defecation – could improve well-being at a low net present cost to the government. Because such public investments are under active public debate in India – where the Prime Minister has announced an ambitious plan to eliminate open defecation by 2019 – these results are of clear policy importance.

Nevertheless, we must acknowledge some important limitations of our analysis. First, because we do not observe the mechanism assigning different Indian districts to improvements in infant mortality and sanitation at different times, and because data limitations force us to use long-term trends in these improvements, we cannot fully confirm the exogeneity of these changes with respect to the outcomes we study. Second, because we are matching census data with survey data decades later, and do not longitudinally track individual children as they become adult workers, we cannot observe mechanisms in a lifetime of health and human capital measurements. However, Spears and Lamba (2016) have recently documented an effect of early-life exposure to open defecation in India on later-childhood cognitive achievement, while Spears (2012b) demonstrates that Indian children who are taller (due in part to better early-life health and net nutrition) also perform better on learning tests. These prior findings suggest that our results are plausible, and that cognitive development is one important mechanism in translating better early-life health into future achievement. Finally, our welfare analysis of quantitative policy implications must assume a unitary household model, because our data source does not allow us to observe the consumption of individual members of the household. Despite these limitations, our results suggest an important role for early-life health in adult economic outcomes in India.

## Figures and Tables

**Table 1:** Summary statistics.

	Mean	SD	25th percentile	75th percentile
Hourly wage (rupees)	10.43	9.11	5.71	12.00
Log of hourly wage (rupees)	2.12	0.62	1.74	2.48
Infant mortality rate in birth year	113.0	41.9	81.3	137.6
Sanitation coverage in birth year	16.8	25.7	0.0	34.0
Birth year	1979.4	4.9	1975	1983
Age in survey	25.6	4.9	22	30
Urban	0.3	0.5	0	1
*n* (adult men)	12,783			

*Notes*: Wages are as reported by the IDHS, either as a reported hourly wage or a computed value from detailed questions about earnings at all jobs and hours worked.

**Table 2:** Main result: Early-life mortality rates and adult wages.

	(1)	(2)	(3)	(4)	(5)
**Panel A: OLS**					
IMR in birth year	−0.00174*	−0.00195*	−0.00166*	−0.00170*	−0.00235**
	(0.000706)	(0.000796)	(0.000742)	(0.000700)	(0.000728)
District fixed effects	✓	✓	✓	✓	✓
Year of birth fixed effects	✓	✓	✓	✓	✓
State × urban fixed effects		✓	✓	✓	✓
Social group × urban indicators		✓	✓	✓	✓
State-specific linear time trends		✓	✓	✓	✓
Job category fixed effects			✓	✓	✓
Individual education indicators				✓	✓
Female literacy in birth year					✓
*n* (adult men)	12,783	12,783	12,783	12,783	11,538

**Panel B: IV (linearly interpolated IMR instrumented with log interpolated IMR)**					
IMR in birth year	−0.00166**	−0.00167*	−0.00139*	−0.00156*	−0.00206**
	(0.000597)	(0.000718)	(0.000696)	(0.000673)	(0.000691)
District fixed effects	✓	✓	✓	✓	✓
Year of birth fixed effects	✓	✓	✓	✓	✓
State × urban fixed effects		✓	✓	✓	✓
Social group × urban indicators		✓	✓	✓	✓
State-specific linear time trends		✓	✓	✓	✓
Job category fixed effects			✓	✓	✓
Individual education indicators				✓	✓
Female literacy in birth year					✓
First-stage F-stat	14,016	4797	4339	3050	3428
*n* (adult men)	12,783	12,783	12,783	12,783	11,538

*Notes*: *p* values: The dependent variable is the log of adult hourly wages in rupees in the IHDS. †=0.1, * =0.05, ** =0.01, ** * =0.001. Standard errors clustered at the district level. Education indicators include a full set of indicators for grade level interacted with literacy. “IMR in birth year” and “female literacy in birth year” both vary at the district-year level. IMR is measured as deaths in the first year of life per 1000 live births.

**Table 3:** No effect on chosen schooling levels.

	(1)	(2)	(3)
	Dependent variable: years of schooling		
IMR in birth year	0.00838		0.0235
	(0.00610)		(0.0175)
Sanitation in birth year		0.00951	0.0206
		(0.0104)	(0.0155)
District fixed effects	✓	✓	✓
Year of birth fixed effects	✓	✓	✓
State × urban fixed effects	✓	✓	✓
Social group × urban indicators	✓	✓	✓
State-specific linear time trends	✓	✓	✓
*n* (adult men)	12,718	12,340	5010

*Notes*: *p* values: †=0.1, * =0.05, ** =0.01, ** * =0.001. Standard errors clustered at the district level. “IMR in birth year” and “sanitation in birth year” both vary at the district-year level.

**Table 4:** Robustness: Adult wages and early-life exposure to open defecation.

	(1)	(2)	(3)	(4)	(5)	(6)
Sanitation in birth year	0.00296***	0.00226	0.00181***	0.00322^†^	0.00276	0.00323^†^
	(0.000509)	(0.001611)	(0.000509)	(0.00169)	(0.00168)	(0.00183)
District fixed effects	✓	✓	✓	✓	✓	✓
Year of birth fixed effects	✓	✓	✓	✓	✓	✓
State × urban fixed effects		✓		✓	✓	✓
State-specific linear time trends		✓		✓	✓	✓
Social group × urban indicators		✓		✓	✓	✓
Job category fixed effects			✓	✓	✓	✓
Individual education indicators					✓	✓
Female literacy in birth year						✓
*n* (adult men)	6134	6134	6134	6134	6134	4664

*Notes*: The dependent variable is the log of adult hourly wages in rupees in the IHDS. *p* values: †=0.1, * =0.05, ** =0.01, ** * =0.001. Standard errors clustered at the district level. Education indicators include a full set of indicators for grade level interacted with literacy. “Sanitation in birth year” and “female literacy in birth year” both vary at the district-year level. Sanitation is the percent of households who use a toilet or latrine, rather than defecate in the open.

**Table 5:** Robustness: Regression of wages on IMR and sanitation for stayers.

	(1)	(2)	(3)	(4)
Sample	Full	Non-migrants	Full	Non-migrants
IMR in birth year	−0.00174*	−0.00162**		
	(0.000706)	(0.000602)		
Sanitation in birth year			0.00296***	0.00255***
			(0.000509)	(0.000528)
District fixed effects	✓	✓	✓	✓
Year of birth fixed effects	✓	✓	✓	✓
*n* (adult men)	12,783	11,017	6134	5533

*Notes*: The dependent variable is the log of adult hourly wages in rupees in the IHDS. *p* values: †=0.1, * =0.05, ** =0.01, ** * =0.001. Standard errors clustered at the district level.

**Table 6:** Comparing coefficients for wages and consumption

	(1)	(2)	(3)	(4)
Dependent variable:	Hourly wage	Consumption	Consumption	Consumption
Sample:	Full	Full	Main earner	Not main
IMR in birth year	−0.00195*	−0.00152*	−0.00173*	0.000211
	(0.000796)	(0.000646)	(0.000780)	(0.00105)
District fixed effects	✓	✓	✓	✓
Year of birth fixed effects	✓	✓	✓	✓
State × urban fixed effects	✓	✓	✓	✓
Social group × urban indicators	✓	✓	✓	✓
State-specific linear time trends	✓	✓	✓	✓
*n* (adult men)	12,783	12,716	8438	4278

*Notes*: *p* values: †=0.1, * =0.05, ** =0.01, ** * =0.001. Standard errors clustered at the district level.

## Footnotes

2 Barreca (2010) also evaluates the long-term economic impact of exposure to malaria in the 20th-century United States, and finds that increased in-utero and postnatal exposure is associated with a potentially substantial but not precisely estimated reduction in income.

3 Note that we therefore ignore any general equilibrium effect whereby labour demand responds, over time, to changes in average human capital. For example, if an improving disease environment in the 1980s caused more factories to be built in a district in the 1990s, this change in labour demand would be absorbed by the district fixed effect in our 2005 cross-section, insofar as it impacted workers of different ages in our sample equally. If such endogenous demand responses are quantitatively important, then the true positive effect of the early-life health environment on wages may be greater than our estimates.

4 In 2005, India was divided into 28 states and 7 union territories; Telangana separated from Andhra Pradesh in 2014 to form the 29th state. States and territories are then subdivided into districts, of which there were 640 as of the 2011 Census of India, for an average district population of around 2 million.

5 In cases where a respondent is not paid an hourly wage, the IHDS computes one from detailed questions about earnings at all jobs and hours worked; see Online Appendix B.1 for more discussion of the IHDS wage variable.

6 In a similar empirical strategy, Almond et al. (2012) have recently used state-by-year variation in infant mortality rates in U.S. states to identify effects of the early-life disease environment on adult health among future mothers.

7 For most districts, the regression had three observations (1981, 1991, 2001), although for a few districts one of these years is missing. The log-linear prediction is done similarly, running district-specific linear regressions of log IMR on year and using the coefficients to predict IMR for each year.

8 Although latrines are a normal good, more likely to be owned by richer people, we are studying district-level sanitation rates, which are not well explained by wealth in South Asia. Even the richest people in India live near people who defecate in the open (Spears, 2013), which would have been even more the case in the 1970s and 1980s. For example, Geruso and Spears (2015) show that, despite the fact that Muslims are notably poorer than Hindus in India on average, the average Muslim lives in a local area where many fewer people defecate in the open than the average Hindu; Muslims are substantially less likely to defecate in the open than Hindus in India (note that we control for religion in our analysis). Similarly, Ghosh et al. (2014) explore consequences of the fact that people in Bangladesh are much less likely to defecate in the open than people in the neighboring Indian state of West Bengal, even though people in West Bengal are much richer, on average. Exposure to open defecation in South Asia is not a mere proxy for socioeconomic status.

9 Rural open defecation in the 2011 census remains high at 70%.

10 The seven categories are family (own) farm work, animal care, agricultural wage labour, non-agricultural wage labour, a salaried job, work at a family business, and any other work (the omitted category).

11 Even so, Cutler et al. (2010) also document that, in the 1991 Census, only 7.5% of all rural residents live outside their district of birth.

12 Cameron and Miller (2015) propose a strategy for two-dimensional clustering of standard errors: in our case, by districts, and by age cohorts. When we implement this procedure, the standard error in IMR *decreases*, although the number of birth cohorts in our study may be too small for credible asymptotic estimates. We will not further discuss multi-dimensional clustering, and note here merely that this procedure gives no reason to suspect that our standard errors are too small.

13 In fact, additional results available upon request indicate that early-life IMR exposure did not significantly affect membership of our categories. This finding is contrary to that in Vogl (2014) for Mexico, where part of the height premium is due to the fact that taller individuals sort into occupations with higher intelligence requirements.

14 As a sensitivity analysis to rule out our results being driven by a correlation between structural poverty and local labour markets, an anonymous referee proposed that we re-run our regressions after dropping the 5% poorest districts. The results from this exercise are extremely similar for both the IMR regressions and the sanitation regressions in Section 5.1, and are available upon request.

15 Of course, this is not evidence against the well-established fact that sanitation is an important determinant of infant mortality; nothing in our analysis is designed as an identification strategy to isolate the effect of open defecation on IMR (which is the independent variable), and other factors that shape mortality would also have been changing over this period. Additionally, rural open defecation would have been almost universal and therefore only very slowly changing before 1981 (the data when we start using sanitation data), so a lack of correlation with IMR is unsurprising.

16 Lee et al. (1997) discuss the possibility that selection in a high-mortality environment can bias results towards an understatement of the effect of sanitation improvements, both because of selection (improved sanitation leads to increased survival of unhealthy children) and because of a reduced allocation of household resources to children. We do not control for this effect, and so our results are likely to be underestimates of the effect of IMR and sanitation on wages, if this bias is important.

17 The full set of quantities used is available upon request.

18 It would require a fairly complicated migration pattern: it would have to be the case that higher-skill individuals among the younger cohorts net migrate selectively into districts where the mortality environment improved the most, or out of districts where the mortality environment did not improve. Therefore, wages among young workers in improved districts would be higher not because of any causal effects of the improved disease environment but rather because they are naturally more skilled workers. Alternatively, our estimates could be biased if older high-skill workers net migrated to districts that did not see significant improvements in mortality environment.

19 Because we only have sanitation data for very young workers, it is not feasible to estimate the gradient between sanitation coverage and consumption, as young workers play a small role in shaping their household's consumption.

20 Several anonymous referees have suggested that these results could reflect a selection mechanism, in which different early-life disease environments could affect the probability of an individual becoming the main earner in their household. However, such a mechanism should bias our results away from finding a positive effect on main earners: if lower IMR raises a worker's earning potential and thus makes them more likely to be their household's main earner, this should make more low-skill individuals main earners, lowering the average wage of main earners. Our results do not give any indication that this has happened.

21 See, for example, the survey of results provided in Table 9 of Fonseca and Ventosa-Santaulària (2011). This is very likely to hold in India as well, given that our quantile regressions in Online Appendix F indicate that the impact is larger at higher incomes, where people are most likely to be paying taxes.

22 We use an exchange rate of $1 US = 54.5481 rupees, which was the average during the 2012–13 fiscal year.

23 This is another conservative assumption, as we ignore economic growth and population growth, and in particular we ignore the possibility that younger cohorts, being better educated, earn more even in the business-as-usual scenario.

24 3.81% was the real interest rate facing the Indian government as of February 2014: an 8.86% interest rate on 10-year bonds minus 5.05% inflation.

25 Alternatively, present-value government revenue increases by about $1.2 billion in response to a reduction in IMR by one point on demographers’ traditional scale of deaths per thousand births.

26 Specifically, 481,743,311 out of a total population of 1,210,569,573 are recorded as workers of some kind, for an employment rate of 39.79%.

Appendix A A supplementary appendix associated with this article can be found, in the online version, at http://dx.doi.org/10.1016/j.ehb.2015.11.006.

## References

[bib0005] Acemoglu D., Johnson S. (2007). Disease and development: the effect of life expectancy on economic growth. J. Polit. Econ..

[bib0010] Alderman H., Behrman J.R. (2006). Reducing the incidence of low birth weight in low-income countries has substantial economic benefits. World Bank Res. Obs..

[bib0015] Alderman H., Hoddinott J., Kinsey B. (2006). Long term consequences of early childhood malnutrition. Oxford Econ. Papers.

[bib0020] Alderman H., Hoogeveen H., Rossi M. (2009). Preschool nutrition and subsequent schooling attainment: longitudinal evidence from tanzania. Econ. Dev. Cult. Change.

[bib0025] Almond D., Currie J., Herrmann M. (2012). From infant to mother: early disease environment and future maternal health. Labour Econ..

[bib0030] Baird S., Hicks J.H., Kremer M., Miguel E. (2011). Worms at work: Long-run impacts of child health gains.

[bib0035] Barreca A.I. (2010). The long-term economic impact of in utero and postnatal exposure to malaria. J. Hum. Resour..

[bib0040] Bleakley H. (2007). Disease and development: evidence from hookwork eradication in the American South. Q. J. Econ..

[bib0045] Bleakley H. (2010). Health, human capital, and development. Annu. Rev. Econ..

[bib0050] Bleakley H. (2010). Malaria eradication in the Americas: a retrospective analysis of childhood exposure. Am. Econ. J.: Appl. Econ..

[bib0055] Bozzoli C., Deaton A., Quintana-Domeque C. (2009). Adult height and childhood disease. Demography.

[bib0060] Cameron A.C., Miller D.L. (2015). A practitioner's guide to cluster-robust inference. J. Hum. Resour..

[bib0065] Case A., Paxson C. (2008). Stature and status: height, ability, and labor market outcomes. J. Polit. Econ..

[bib0070] Checkley W., Buckley G., Gilman R.H., Assis A.M., Guerrant R.L., Morris S.S., Mølbak K., Valentiner-Branth P., Lanata C.F., Black R.E. (2008). Multi-country analysis of the effects of diarrhoea on childhood stunting. Int. J. Epidemiol..

[bib0075] Currie J. (2009). Healthy, wealthy, and wise: socioeconomic status, poor health in childhood, and human capital development. J. Econ. Lit..

[bib0080] Currie J., Vogl T. (2013). Early-life health and adult circumstance in developing countries. Annu. Rev. Econ..

[bib0085] Cutler D., Fung W., Kremer M., Singhal M., Vogl T. (2010). Early-life malaria exposure and adult outcomes: evidence from malaria eradication in India. Am. Econ. J.: Appl. Econ..

[bib0090] Cutler D., Miller G. (2005). The role of public health improvements in health advances: the twentieth-century united states. Demography.

[bib0095] de Oliveira V.H., Quintana-Domeque C. (2014). Early-life environment and adult stature in brazil: an analysis for cohorts born between 1950 and 1980. Econ. Hum. Biol..

[bib0100] Deaton A., Arora R. (2009). Life at the top: the benefits of height. Econ. Hum. Biol..

[bib0105] Desai S., Dubey A., Joshi B., Sen M., Shariff A., Vanneman R. (2007). India Human Development Survey (ihds). Computer file, icpsr22626-v2.

[bib0110] Esrey S.A., Habicht J.-P., Casella G. (1992). The complementary effect of latrines and increased water usage on the growth of infants in rural Lesotho. Am. J. Epidemiol..

[bib0115] Fonseca F.J., Ventosa-Santaulària D. (2011). Revenue elasticity of the main federal taxes in mexico. Latin Am. J. Econ..

[bib0120] Galiani S., Gertler P., Schargrodsky E. (2005). Water for life: the impact of the privatization of water services on child mortality. J. Polit. Econ..

[bib0125] Geruso M., Spears D. (2015). Neighborhood sanitation and infant mortality.

[bib0130] Ghosh A., Gupta A., Spears D. (2014). Are children in West Bengal shorter than children in bangladesh?. Econ. Polit. Wkly..

[bib0135] Glewwe P., Jacoby H.G., King E.M. (2001). Early childhood nutrition and academic achievement: a longitudinal analysis. J. Public Econ..

[bib0140] Hansen C.W. (2014). Cause of death and development in the US. J. Dev. Econ..

[bib0145] Hatton T.J. (2014). How have Europeans grown so tall?. Oxford Econ. Paper.

[bib0150] Humphrey J.H. (2009). Child undernutrition, tropical enteropathy, toilets, and handwashing. Lancet.

[bib0155] Kov P., Smets S., Spears D., Vyas S. (2013). Growing taller among toilets: Evidence from changes in sanitation and child height in Cambodia, 2005–2010..

[bib0160] Lee L., Rosenzweig M.R., Pitt M.M. (1997). The effects of improved nutrition, sanitation, and water quality on child health in high-mortality populations. J. Econom..

[bib0165] Lin A., Arnold B.F., Afreen S., Goto R., Huda T.M.N., Haque R., Raqib R., Unicomb L., Ahmed T., J.M.C., Luby S.P. (2013). Household environmental conditions are associated with enteropathy and impaired growth in rural Bangladesh. Am. J. Trop. Med. Hyg..

[bib0170] Rosenzweig M.R., Stark O. (1989). Consumption smoothing, migration, and marriage: evidence from rural India. J. Polit. Econ..

[bib0175] Spears D. (2012). Effects of rural sanitation on infant mortality and human capital: Evidence from a local governance incentive in India.

[bib0180] Spears D. (2012). Height and cognitive achievement among Indian children. Econ. Hum. Biol..

[bib0185] Spears D. (2013). How much international variation in child height can sanitation explain? Policy Research Working Paper no. 6351.

[bib0190] Spears D., Lamba S. (2016). Effects of early-life exposure to rural sanitation on childhood cognitive skills: evidence from India's total sanitation campaign. J. Hum. Resour..

[bib0195] Unicef (2012). Infant and Child Mortality in India: Levels, Trends, and Determinants.

[bib0200] Unicef and WHO (2012). Progress on Drinking Water and Sanitation: 2012 Update. Joint Monitoring Programme for Water Supply and Sanitation.

[bib0205] Vogl T. (2014). Height, skills, and labor market outcomes in Mexico. J. Dev. Econ..

[bib0210] Watson T. (2006). Public health investments and the infant mortality gap: Evidence from federal sanitation interventions on u.s. Indian reservations. J. Public Econ..

