# Supplementary material for: What doesn't kill you makes you poorer: Adult wages and early-life mortality in India
Source: Econ Hum Biol. 2016 May;21:1–16. doi: 10.1016/j.ehb.2015.11.006 (PMC4937106; doi:10.1016/j.ehb.2015.11.006)
Supplement: Supplementary file 1 [file mmc1.pdf]

For Online Publication

Supplementary Online Appendix for: What Doesn't Kill You Makes You Poorer: Adult Wages and Early-Life Mortality in India

Nicholas Lawson
Aix-Marseille University (Aix-Marseille School of Economics), CNRS & EHESS

Dean Spears
Centre for Development Economics, Delhi School of Economics

November 6, 2015

A Introduction

In this supplementary online appendix, we present additional discussion, empirical results and fiscal and welfare calculations. References to numbered equations, tables, etc. refer to the numbers from the article, and the numbering carries on from where it left off there.

We begin with a discussion of the wage variable calculated by the IHDS, as well as a list of the Indian states represented in the data, in section B, followed by an evaluation of the evidence for selective migration in section C. Section D then presents a robustness check that shows that our estimates are not sensitive to the start date of our data. Section E presents the calculations used to derive the main fiscal and welfare results in section 6 of the article. Section F then presents the results of quantile regressions of wages and consumption on IMR and sanitation, and section G uses the results of these regressions to provide robustness checks for the fiscal and welfare analyses in section 6.

B The IHDS Data

This appendix presents a brief discussion of a few features of our data. First, we provide more detail about the wage data used in our analysis. Subsequently, we present a list of the 17 Indian states represented in our data.

B.1 The IHDS Wage Variable

In our estimation, the main outcome variable is the hourly wage calculated by the IHDS as "WS8HOURLY." In order for IHDS to report a wage, individuals must have recorded at least one job, which is the case for 49% of men aged 18 to 34; this constitutes our sample. Of the rest, approximately half report working less than 240 hours in the year, which suggests that most are students. Among the 25% of the 18-34 sample that report working 240 hours or more but without a formal job, 65% work on a family farm or in family animal husbandry; without wage data for these farm labourers, we are unable to include them in our sample.

Note that any concern about sample selection would be expected to work against finding our result: if experiencing a better early-life disease environment makes marginal individuals more likely to engage in paid employment, we would expect to find more low-skill workers in our sample, lowering the average wage received by individuals born in a better disease environment. Thus, our results may be underestimates if this selection is important.

B.2 Data Coverage: Indian States

Table 7 presents the 17 states covered by our data, along with the number of observations in our data, and each state's population and share of the total Indian population. As noted in section 3.1, our data covers the 13 most-populated states as of the 2011 Census, comprising over 85% of the total population.

C No Evidence of Selective Migration

Our results in Table 5 indicate that selective migration does not appear to be responsible for our results. In this appendix, we further test to see if there is any evidence for selective migration at all.

We divide the sample into two halves, with all districts above the median improvement in IMR in our sample period in one half ("improved districts") and all districts below the median in the other ("non-improved districts"), and calculate total migrants in each half by age. Then we examine the share of overall migrants going to improved districts by age. If selective migration was occurring, we might expect that a greater share of migrants would be found in improved districts among the younger cohorts, but in Figure 5 we see no evidence of this.

Next, we also divide our population into two halves: we regress wages on year-of-birth and district fixed effects, and place all individuals with above-median residuals in one half and those below the median in the other, as a proxy for high- and low-skill groups. We then ask: has there been more high-skilled migration into improved districts relative to less-improved districts among young individuals? The results are presented in Figure 6, and once again there is no evidence for a selection of migrants into improved districts; in fact, if anything, the trend is in the opposite direction: high-skilled migration as a fraction of total migration towards improved districts is lower for younger workers. Therefore, we can conclude that selective migration is not a problem for our analysis, because it does not appear to have occurred.

D Sensitivity Analysis on Start Date

Our analysis using infant mortality rates is based on data on individuals born in 1971 and later, but the first district-level data on IMR is not available until the 1981 Census. Therefore, as described in section 3, we run district-specific regressions of Census IMR data and use the coefficients to predict IMR for each district from 1971 to 1989. As a result, the IMR observations used as the independent variable in our main regressions is partly an interpolation between Census rounds, and partly an extrapolation into the past. We confirm in this appendix that this specification does not bias our results.

Consider our main result in column 1 of panel A of Table 2; we can re-run this regression dropping the early years of our sample, and Figure 7 presents the results. As the graph moves from left to right, we truncate our sample by including only adult men born after the numbered year. Thus, the first estimate is from our actual sample, the last estimate omits

all men born before 1981 (and thus every observation with a backwards-projected IMR), and the years progress horizontally from one extreme to the other.

The most obvious consequence of reducing the sample size as the estimates move from left to right is the increase in the standard error and confidence interval: estimates with a smaller sample size will always be less precise, all else equal. The second consequence is that, if anything, our point estimate becomes larger in absolute value as the sample is restricted. This difference is slight and should not be overemphasized; however, it is consistent with the possibility of some attenuation in our pre-1980 births. Nothing in these results – including completely excluding 1970s births – suggests that our main result is an overestimate of the association between early-life IMR and adult wages.

E Fiscal and Welfare Calculations

In this appendix, we provide the details of the calculations of the fiscal and welfare gains from improved early-life health discussed in section 6.

E.1 Fiscal Calculations

As discussed in section 6.1, it is assumed that each cohort produces \$2.34 billion of tax revenues per year in the baseline scenario. Therefore, a 1% point reduction in the infant mortality rate raises tax revenues by 1.74% from each treated cohort, or about \$40.73 million per cohort per year. These gains begin 18 years from now when the first treated year-of-birth cohort enters the labour market, and will continue to phase in until 57 years from now when all workers in the labour market will have received the treatment, at which point revenues will be \$1.63 billion higher than in the business-as-usual scenario. The revenue gains will end 157 years from now when the last treated cohort leaves the labour market.

Our calculation, therefore, is the following:

$$G = \sum_{t=18}^{157} \left(\frac{1}{1+r} \right)^t \min\{t-17, 158-t, 40\} g_y$$

where G is the increase in present-value tax revenues, $r = 0.0381$ is the discount rate, and $g_y = 40.73$ is the revenue gain per cohort per year. For the sensitivity analyses in panel A of Figure 3, r and g_y are simply replaced with the appropriate values in each case.

When considering the elimination of open defecation, we assume that each percentage point reduction in open defecation raises average wages by 0.296% from the current level, and thus raises tax revenues by \$6.93 million per cohort per year. The revenue gains begin 18 years from now, with a gain of \$368 million from the first treated cohort, or 53.1 times \$6.93 million; however, each subsequent cohort produces a smaller revenue gain because the counterfactual involves open defecation declining at a rate of 1.05% per year. There are 51 treated cohorts, and each cohort $c \in \{1, ..., 51\}$ produces a present-value revenue gain equal to:

$$G_c = \sum_{t=c+17}^{c+56} \left(\frac{1}{1+r}\right)^t (54.15 - 1.05c) g_y$$

where $g_y = 6.93$ is the revenue gain per cohort per year from a 1% point reduction in open defecation. Adding up the present-value revenue gains from all 51 treated cohorts produces the final result of \$60.48 billion in the baseline case, and replacing r and g_y with the appropriate values allows us to generate the sensitivity analyses in panels B and C of Figure 3.

E.2 Welfare Calculations

Given the assumptions made in section 6.2, each 1% point reduction in IMR is associated with a \$47.76 increase in annual after-tax income for the average worker; we assume that this translates into an equal increase in his household's consumption. Given an estimated population of workers of about 481 million, the increase in consumption per cohort per year is \$575 million, and we add up the consumption gains over 157 years as in the fiscal analysis above:

$$G = \sum_{t=18}^{157} \left(\frac{1}{1+r}\right)^t \min\{t - 17, 158 - t, 40\} g_y$$

where G is now our estimate of the welfare gain, and $g_y = 575$ now represents the consumption gain per cohort per year. We find a present-value increase in welfare of \$165.15 billion in the baseline case. For the sensitivity analyses in panel A of Figure 4, r and the increase in annual consumption per worker are simply replaced by the appropriate values.

For the consumption regressions, as stated in section 6.2 we find that a 1% point decline in IMR is associated with a \$7.93 increase in consumption per person per year. The 2011 Census

found about 362 million main workers in India, so we assume that each main worker supports 3.34 individuals (total population divided by main workers). This implies a consumption gain per cohort per year of work of \$240 million, and adding these gains exactly as above produces a present-value increase in welfare of \$68.91 billion.

Finally, our sanitation calculations begin with the fact that a 1% point reduction in open defecation is associated with a \$9.07 per year increase in after-tax income for an average worker; again, we assume an equal increase in his household's consumption. For each cohort c, we can add up each worker's total discounted gains from the elimination of open defecation:

$$G_c = \sum_{t=c+18}^{c+57} \left(\frac{1}{1+r}\right)^t \max\{54.15 - 1.05c, 0\} g_y$$

where $g_y = \$9.07$; this leads to a total of \$4653 for someone with $c = 0$, i.e. someone born today. Simply adding up G_c from each cohort, we find a total present-value after-tax income gain of \$853.99 billion, and the sensitivity analyses in panels B and C of Figure 4 are produced in the usual way.

F Quantile Regressions

In this appendix, we ask the question: how does the association between the early-life disease environment and wages or consumption vary across the ability distribution? The answer to this question will help us to further understand the way in which the disease environment interacts with other personal characteristics, and it is also of some importance for our fiscal and welfare analysis. If the impact is concentrated on high-ability (and thus typically high-income) individuals, for example, the effect on tax revenues is likely to be larger than would otherwise be the case, whereas the welfare impact may be smaller because of increased inequality.

We can answer this question using quantile regressions. Let us return to our original regression equation:

$$\ln(y_{idt}) = \beta \, disease_{dt} + X_{idt}\theta + \varepsilon_{idt}$$

where $disease_{dt}$ is either IMR or sanitation, and X_{idt} is now a general set of controls, possibly including the year and district fixed effects. For any percentile τ, the typical quantile

regression[1] estimates the following:

$$\ln(y_{idt}) = \beta_\tau disease_{dt} + X_{idt}\theta_\tau + \varepsilon_{idt}.$$

where both β and θ are allowed to vary across the distribution. However, we prefer to think of the fixed effects and time trends contained in X_{idt} as constant additive components, which therefore would not vary across the distribution; therefore, we use the two-step procedure in Canay (2011) to estimate the following:

$$\ln(y_{idt}) = \beta_\tau disease_{dt} + X_{idt}\theta + \varepsilon_{idt}.$$

Canay's procedure is simple: we perform an OLS regression of $\ln(y_{idt})$ (wages or consumption) on $disease_{dt}$ and a set of controls X_{idt}, and calculate $\ln(\hat{y}_{idt}) = \ln(y_{idt}) - X_{idt}\hat{\theta}$, where $\hat{\theta}$ is the estimated coefficient on X_{idt}. Then we perform a quantile regression of $\ln(\hat{y}_{idt})$ on $disease_{dt}$. The results are displayed in panels A through C of Figure 8, for the case in which X_{idt} contains only district and year-of-birth fixed effects; the blue bands represent 95% confidence intervals using bootstrapped standard errors.

The results are suggestive of a larger effect of the disease environment on wages towards the upper end of the distribution; the consumption estimates are flat and never attain statistical significance. However, the next question is: which distribution are we looking at? That is, which variable is distributed along the x-axis? Because the regressions condition on $disease_{dt}$ and X_{idt}, the distribution in question is the distribution of $\ln(y_{idt})$ controlling for $disease_{dt}$ and X_{idt}, which is equivalent to the distribution of ε_{idt}; we are looking at the effect of the disease environment on wages and consumption at different points in the distribution of unobservables. Essentially, we are looking at the effect on individuals based on their place in the distribution of wages in their district and year of birth.

These results remain of interest, because although high and low-income workers are mixed together in the distribution of ε_{idt}, it is still true that on average, an individual who is near the top of the distribution of unobservables will have a higher wage than someone near the bottom of the distribution. There are a few alternative strategies that we can use, however, that might allow us to say something about the effect along the current income distribution;

[1] For example, what we call the typical quantile regression is what `qreg` estimates in Stata.

one is to attempt to transform the coefficients by quantile on the distribution of ε_{idt} into average coefficients by quantile on the distribution of $\ln(y_{idt})$. That is, by estimating the OLS regression of $\ln(y_{idt})$ on $disease_{dt}$ and X_{idt}, we can use the residuals as a proxy for ε_{idt}; if we then make a rank-preservation assumption, implying that a given individual would be at the same position in the distribution of ε for any value of the treatment, we can assign each individual a coefficient β_i based on their place in the distribution of residuals. Then, we can look at the distribution of β_i across the wage or consumption distribution. In panels D through F of Figure 8, we present the smoothed local means of β_i at quantiles of the wage or consumption distributions; given the ad-hoc nature of the calculation, we have not attempted to calculate standard errors, but the means confirm the results so far: the largest effects of the early-life disease environment on wages occur towards the upper end of the distribution. The same appears to be true of the consumption regression as well, though the lack of precision in the quantile estimates make the results less clear than in the wage case.

An alternative strategy is to remove some of the controls, and so in Figure 9, we present the results of quantile regressions without the district and year-of-birth fixed effects. The results are broadly similar to those in Figure 8, though the coefficients are larger, since they are now picking up part of the district and year fixed effects; the consumption estimates are also somewhat altered, in that they now show a larger effect near the bottom of the distribution. However, this approach is not entirely satisfactory, as the regression equation is now almost certainly misspecified, as the omitted fixed effects are surely correlated with both the disease environment and wages.

Finally, Figure 10 presents the results from quantile regressions with the full set of controls: district and year-of-birth fixed effects, but also state $\times$ urban fixed effects, state time trends, and social group $\times$ urban indicators. The estimates are quite similar to those in Figure 8, though somewhat larger and more significant for consumption and smaller and less significant for sanitation.

G Fiscal and Welfare Calculations from Quantile Estimates

In this appendix, we produce alternative estimates of the fiscal and welfare impacts of improved sanitation and early-life disease environment, examining the robustness of our estimates using the results of our main quantile regressions from Figure 8.

G.1 Fiscal Externalities

In appendix F, we discussed how our quantile regression estimates of the effect of IMR and sanitation on wages could be used to assign each individual in our sample an estimated coefficient. We now make use of these personalized coefficients, as calculated in the baseline quantile regressions presented in Figure 8, to produce new estimates of the fiscal effects of the sanitation and disease environment.

Our baseline fiscal and welfare analysis is conducted using 2012-13 dollars, and so we first rescale the incomes in our sample to be representative of the average in 2012. According to the World Bank, GNI per capita in India in 2012 was 83026.8 INR; therefore, we multiply all incomes by a constant to make the mean in our sample 83026.8. Then, to account for the fact that we have a sample of workers, we divide incomes by the ratio of workers to total population.[2]

Then we apply income and consumption taxes to our sample according to 2012-13 tax rules. For income taxes, we apply the official income tax brackets; however, our calculations in the IMR case imply that about 13.7% of the total population of India have incomes above the income tax threshold, but in December 2012 the Finance Minister of India stated that 2.89% of the population paid income taxes. Therefore, for a conservative estimate of fiscal implications accounting for possible tax evasion, we use the Finance Minister's figure: we assume that tax evasion is random with respect to income, and when we shift IMR or sanitation, we charge each individual in our sample with an increase in income tax payments that is around 20% of their increase in official tax liabilities. Then, for consumption taxes,

[2] According to the 2011 Census, this is $\frac{481743311}{1210569573}$; this makes the mean income in our sample equal to the mean income in the actual population of income-earners.

we use the 12% CENVAT tax and apply a tax at rate $\frac{0.12}{1.12}$ on after-tax wages.[3]

The results are as follows. First of all, a 1% point reduction in IMR is associated with an increase in yearly tax revenues of $98.35 million per treated year-of-birth cohort, incorporating $15.38 million of income taxes and $82.97 million of VAT; adding up over 157 years and discounting produces a present-value total of $28.24 billion, or more than twice the value from the baseline analysis. Given that the quantile regressions indicated that the effects on wages were larger towards the upper end of the distribution, where people are more likely to be paying taxes, this is not surprising.

When we consider the elimination of open defecation, the analysis becomes a bit more complicated, because the open defecation rate for our sample is well above 53.1%. Therefore, we assume that "eliminating open defecation" means reducing open defecation by 53.1% points on average (some districts are already relatively close to zero open defecation, so their rate will drop less). The resulting per-year increase in tax revenues for the first treated cohort is $1039.64 million, incorporating $175.67 million of income taxes and $863.96 million of VAT. Then, assuming that gains from "eliminating open defecation" decline linearly over time as in the baseline analysis, we find that the total present-value increase in tax revenues is $170.88 billion, or $1304 per household that stops defecating in the open, nearly 3 times as large as in the baseline analysis.

Therefore, our quantile estimates indicate that our finding of substantial fiscal gains associated with improved early-life disease environment is robust; if anything, our baseline estimates are likely underestimates, because they do not account for the fact that the income gains from an improved early-life disease environment appear to be disproportionately experienced on the upper end of the income distribution.

G.2 Welfare Gains

We can also estimate the welfare impact of both IMR and sanitation using the estimates from our quantile regressions from Figure 8. However, first we need to consider how to account for heterogeneity when making welfare calculations; to do so, we present a basic

[3]Some consumption items are taxed at higher or lower rates, but in the absence of detailed information on their rates and share in consumption, we assume a flat rate on all consumption.

model of individual utility, which we solve for a simple expression for welfare which depends only on empirically estimable coefficients, in the spirit of the "sufficient statistics" method of policy analysis discussed in Chetty (2009). As in section 6, it should be noted that the analysis that follows is based on the assumption of a unitary agent, an assumption that is unlikely to be true in India, particularly when talking about household consumption. Thus, our measure of utility or welfare should be interpreted as an approximation, or as a measure of potential household well-being.

Consider the case of sanitation, and start with the assumption that individuals today receive some utility from toilet or latrine use, denoted by $d \sim F(d)$, which could be positive or negative. Then the fraction L of people who use a latrine is defined by $L = 1 - F(0)$, and we assume that for a cost of p per unit, the government can shift L upwards; essentially, we assume that through direct provision of a latrine and/or education or other activities to raise people's tastes for latrine use, the government is able to take some individuals with negative d and shift them to indifference. In so doing, we ignore any welfare gains of this education on the utility of inframarginal latrine users; for example, while it is possible that community education programs could make people currently using a latrine feel happier about doing so, we have no way to measure this impact and so we ignore it. Notice that this formulation is entirely consistent with a more agnostic view in which we consider changes in IMR: suppose that L is the infant survival rate (1 minus IMR), and the government has some technology with which they can raise L at a cost of p per unit.

We ignore any changes in taxes today, by assuming that if the tax rate needs to change to balance the budget, it will do so in the future for the taxes applied to the affected generations. The result of this is that we can ignore the utility impact of this sanitation/early-life health investment on the current generation, and focus only on the gains to future generations from higher wages and consumption. Begin by considering a single cohort of individuals born today, each facing the average value of L. Assume that each individual receives an income y_i which is constant over time and is the sum of an idiosyncratic component $x_i \sim G(x)$ and a component that depends directly on L: $y_i = x_i + \alpha L$. Individuals obtain utility from consumption according to a CRRA utility function, $U = \frac{c^{1-\theta}}{1-\theta}$, and the government

levies a tax on income $T(y)$ and provides a lump-sum transfer b.[4] Consumption is thus $c = y - T(y) + b$, and if we assume that the budget constraint is balanced through changes in b, the effect of changing L on utility is:

$$\frac{dU}{dL} = c^{-\theta}\left[(1 - t(y))\gamma y + \frac{db}{dL}\right]$$

where $t(y)$ is the marginal tax rate, and $\gamma = \frac{\alpha}{y} = \frac{\frac{dy}{y}}{dL}$ is the percentage increase in wages for a unit increase in L, as estimated earlier. Now consider that the cohort in question begins working at time t_0 and leaves the labour market at time t_1, and that future utilities are discounted using some discount factor β. Then, using a utilitarian social welfare function $V = \sum_{t=t_0}^{t_1} \beta^t E(U)$, the total welfare impact on this cohort is:

$$\frac{dV}{dL} = B(t_0, t_1)E\left(c^{-\theta}\left[(1 - t(y))\gamma y + \frac{db}{dL}\right]\right)$$

where $B(t_0, t_1) = \sum_{t=t_0}^{t_1} \beta^t = \beta^{t_0}\frac{1-\beta^{t_1-t_0+1}}{1-\beta}$ is the sum of discount factors over time. Finally, to put this in dollar terms, normalize by the average marginal utility $E\left(c^{-\theta}\right)$:

$$\frac{dW}{dL} \equiv \frac{\frac{dV}{dL}}{E\left(c^{-\theta}\right)} = B(t_0, t_1)\frac{E\left(c^{-\theta}\left[(1 - t(y))\gamma y + \frac{db}{dL}\right]\right)}{E\left(c^{-\theta}\right)}.$$

For purposes of illustration, suppose the cohort under consideration is the only one affected by the intervention, and the only one for whom b changes. Then the government's budget constraint is:

$$B(t_0, t_1)E(T(y)) = B(t_0, t_1)b + pL$$

and we can differentiate to find:

$$B(t_0, t_1)\frac{dE(T(y))}{dL} = B(t_0, t_1)\frac{db}{dL} + p$$

and therefore $\frac{db}{dL} = \frac{dE(T(y))}{dL} - \frac{p}{B(t_0,t_1)}$. Then the welfare derivative is:

$$\frac{dW}{dL} = B(t_0, t_1)\frac{E\left[c^{-\theta}(1 - t(y))\gamma y\right]}{E\left(c^{-\theta}\right)} + B(t_0, t_1)\frac{dE(T(y))}{dL} - p.$$

This equation tells us something quite simple; notice that the second term is the discounted sum of increased tax revenues, while the third term is the cost of the intervention. These

[4] In this environment, a tax on consumption is implicitly a tax on income; the point is to account for the fraction of income that is paid in one tax or another.

were the two terms of importance in the analysis of the fiscal impacts earlier. Therefore, to estimate the welfare impact of higher wages, we merely need to add to the net fiscal impact the discounted gains in after-tax income or consumption, weighted by marginal utility if there are any distributional impacts.

For the purpose of this analysis, we will focus on a fiscally neutral improvement in sanitation or IMR; i.e. investments in reducing IMR or improving sanitation that produce tax revenue gains equal precisely to their cost. In the absence of distributional effects, we would simply consider the per-person increase in consumption, exactly as in section 6.2; instead, we consider the weighted average increase in consumption, using $c^{-\theta}$ as weights.

To begin with, consider the regression of wages on IMR; in the same way that we can estimate the increase in income and sales taxes paid for each worker in our sample, we can also estimate their increase in after-tax income. Then, we assume log utility (i.e. $\theta = 1$), and calculate the value of $B(t_0, t_1)\frac{E\left[c^{-\theta}(1-t(y))\gamma y\right]}{E\left(c^{-\theta}\right)}$. We find that workers receive an increase in after-tax income that is equivalent on average to 1176 INR per year, or \$21.56, from a 1% point reduction in IMR. Over a 40-year working life, this provides a present-value gain per person of \$232.48, and the economy-wide value is \$74.47 billion, less than half the size of the baseline result, though this is not surprising given that the gains tend to be stronger at the top end of the distribution, where our adjustment for distribution weights the gains less heavily.

Next, we look at the quantile regression of consumption on IMR. Weighting by marginal utility, a 1% point decline in IMR raises average consumption by a value equivalent to \$9.59 per worker per year (accounting for the gains of those supported by each worker). This is equivalent to \$103.39 per worker in present value, a welfare increase of \$1.25 billion per cohort, and a total economy-wide present-value increase of \$33.12 billion, also smaller than the baseline value.

Finally, the quantile regression of wage on sanitation coverage can be used to estimate the welfare gain from eliminating open defecation, using the same procedure as in appendix G.1. The average first-cohort worker's gain in after-tax income is \$208.05 per year, which is equivalent to \$2244 in present-value terms. Adding up the gains over the next century, the economy-wide present value is \$411.84 billion, again smaller than the baseline result due to

the unequal impact across the distribution.

Therefore, because our quantile estimates feature stronger effects at high incomes, when we adjust for distributional impacts we find smaller welfare gains than in the baseline analysis; however, the welfare gains remain highly economically significant in each case.

References

Canay, I. A., 2011. A simple approach to quantile regression for panel data. Econometrics Journal 14 (3), 368–386.

Chetty, R., 2009. Sufficient statistics for welfare analysis: A bridge between structural and reduced-form methods. Annual Review of Economics 1, 451–488.

Figure 5: Fraction of Migrants in Improved (Top Half) Districts

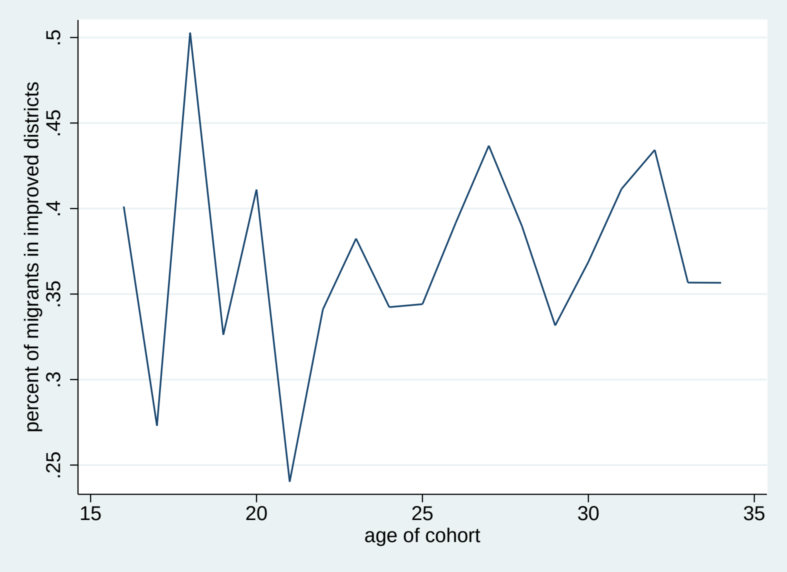

Notes: For the purpose of this figure, our sample of districts has been ordered by our estimates of the district-level decline in IMR from 1971 to 1991; the line represents the fraction of all migrants, for each age group, who are observed in the half of districts with the larger decline in IMR.

Figure 6: Fraction of High- and Low-Skilled Migrants in Improved Districts

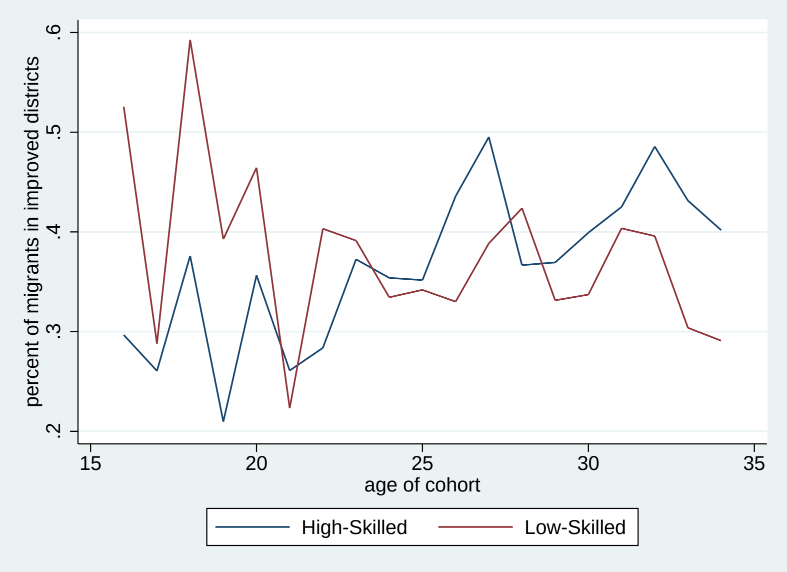

Notes: For the purpose of this figure, our sample of districts has been ordered by our estimates of the district-level decline in IMR from 1971 to 1991, and the sample of individuals split into halves according to their residuals from a regression of wages on year-of-birth and district fixed effects. The two lines represent the fractions of migrants in the half of districts with the larger decline in IMR; the red line corresponds to individuals with below-median residuals, while the blue line represents those with above-median wage residuals.

Figure 7: IMR Coefficient by Start Date

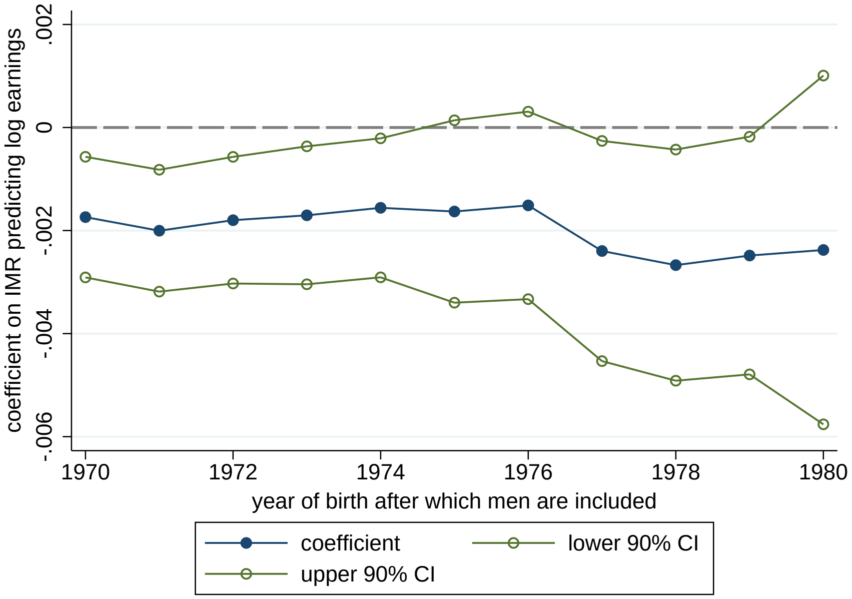

Notes: This figure presents estimated coefficients and 90% confidence intervals for regressions of log of adult wages on IMR, with district and year of birth fixed effects as controls, as in column 1 of panel A of Table 2. The start date of our data increases as we move to the right, with the year on the horizontal axis denoting the year of birth up to which all observations are discarded.

Figure 8: Quantile Regressions with District and Year Fixed Effects

(A) Coefficients from QR of Wage on IMR

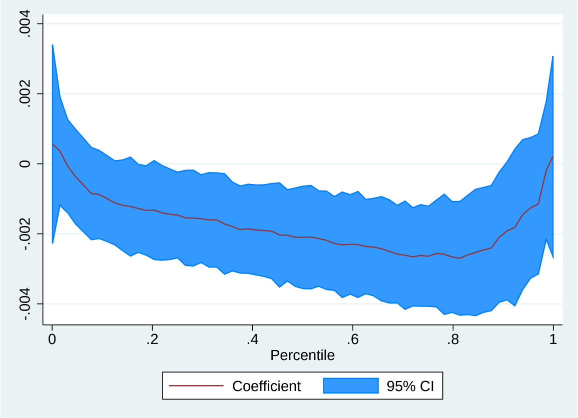

(D) Local Mean Effect of IMR on Wages

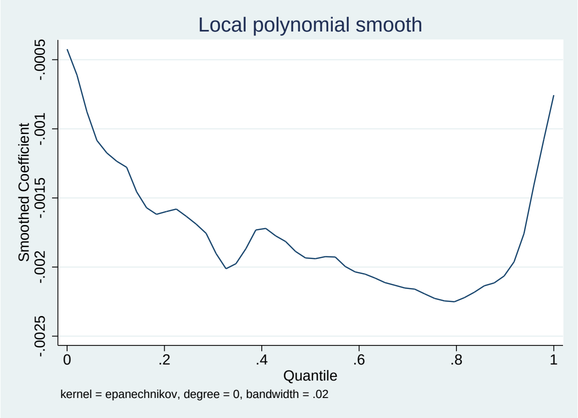

(B) Coefficients from QR of Wage on Sanitation

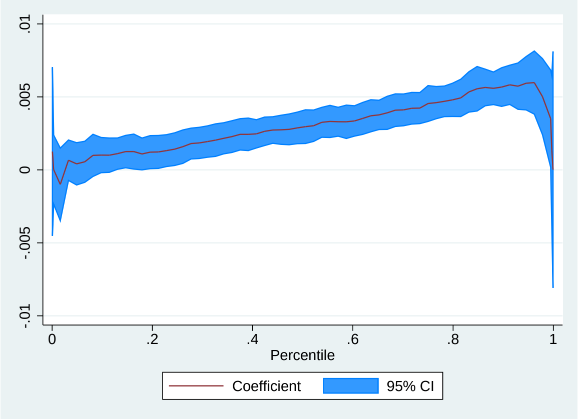

(E) Local Mean Effect of Sanitation on Wages

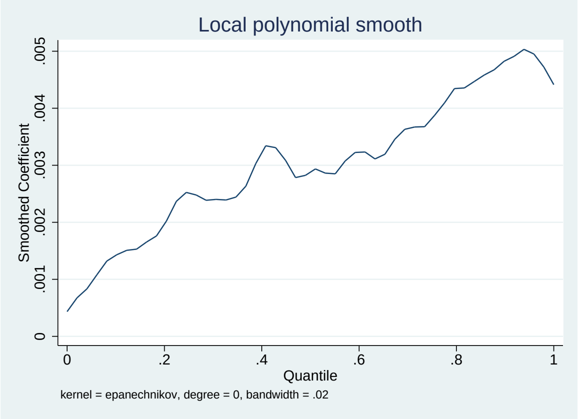

(C) Coefficients from QR of Consumption on IMR

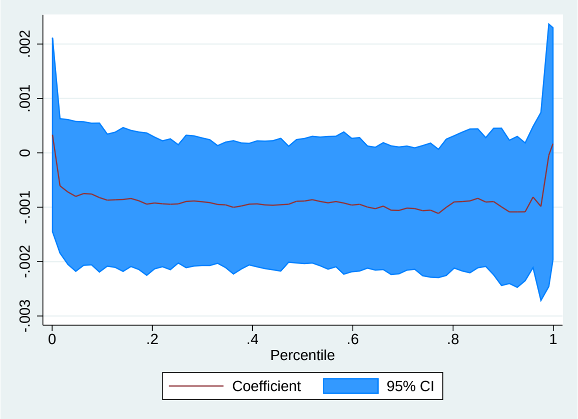

(F) Local Mean Effect of IMR on Consumption

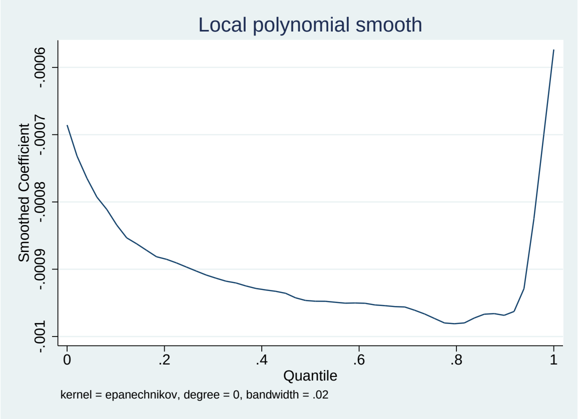

Notes: Panels A through C present quantile regressions with district and year fixed effects using the Canay (2011) procedure. Panels D through F present β_i as a function of the wage or consumption distribution, averaged using a smoothed local mean.

Figure 9: Quantile Regressions with No Fixed Effects

(A) Coefficients from QR of Wage on IMR

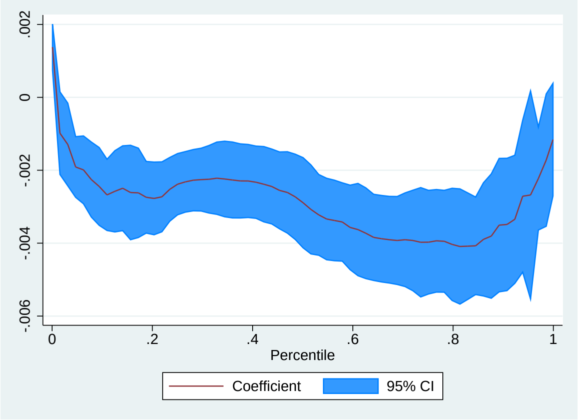

(B) Coefficients from QR of Wage on Sanitation

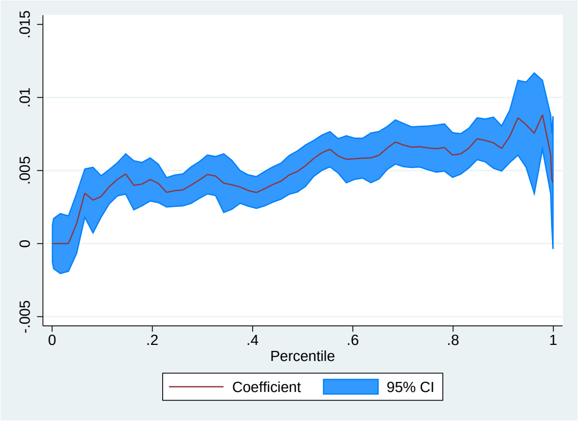

(C) Coefficients from QR of Consumption on IMR

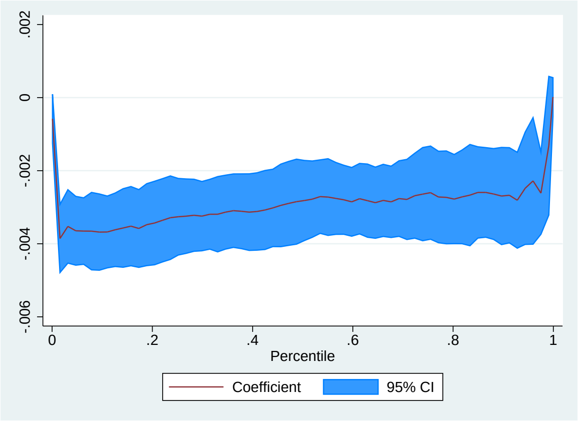

(D) Local Mean Effect of IMR on Wages

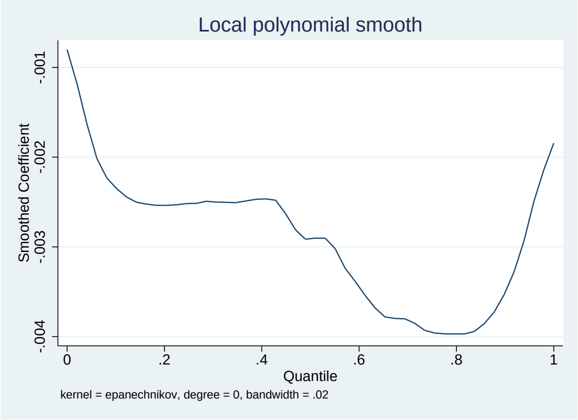

(E) Local Mean Effect of Sanitation on Wages

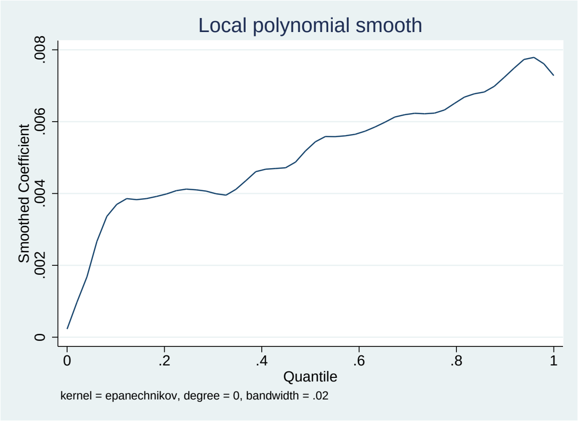

(F) Local Mean Effect of IMR on Consumption

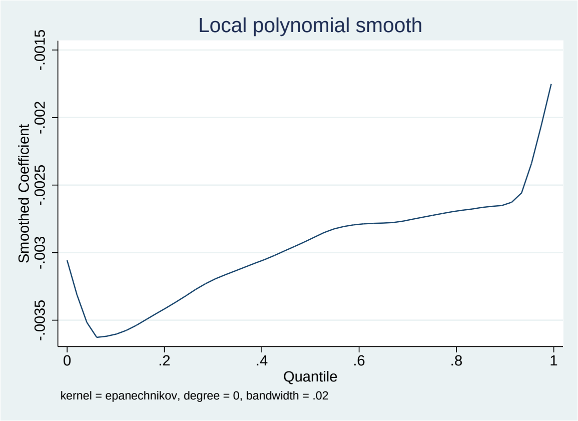

Notes: Panels A through C present unconditional quantile regressions. Panels D through F present β_i as a function of the wage or consumption distribution, averaged using a smoothed local mean.

Figure 10: Quantile Regressions with Full Set of Controls

(A) Coefficients from QR of Wage on IMR

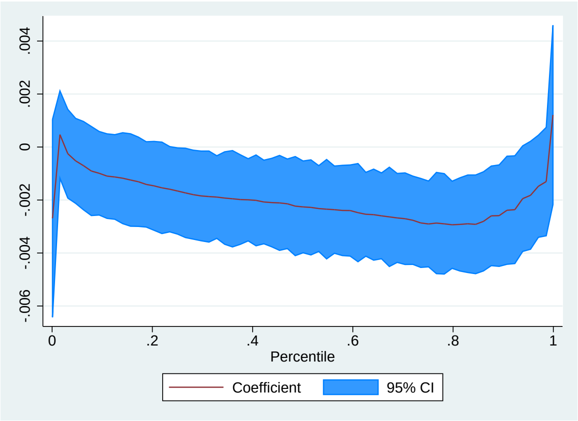

(D) Local Mean Effect of IMR on Wages

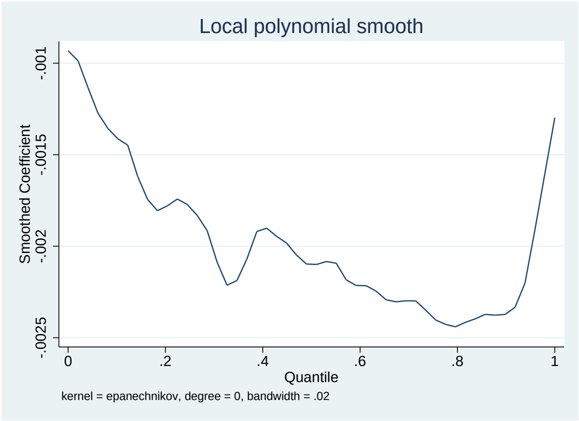

(B) Coefficients from QR of Wage on Sanitation

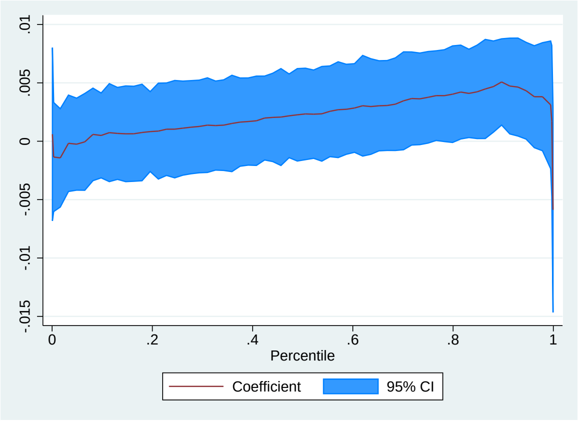

(E) Local Mean Effect of Sanitation on Wages

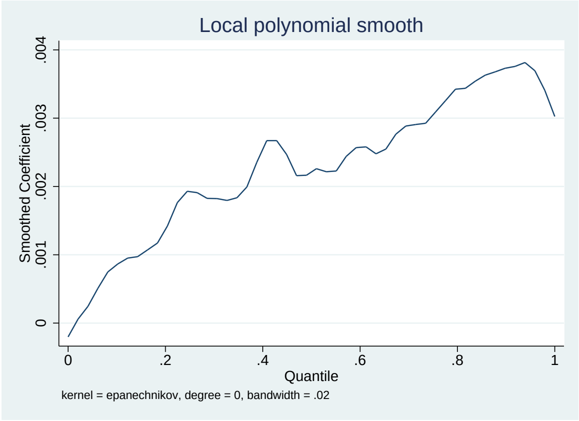

(C) Coefficients from QR of Consumption on IMR

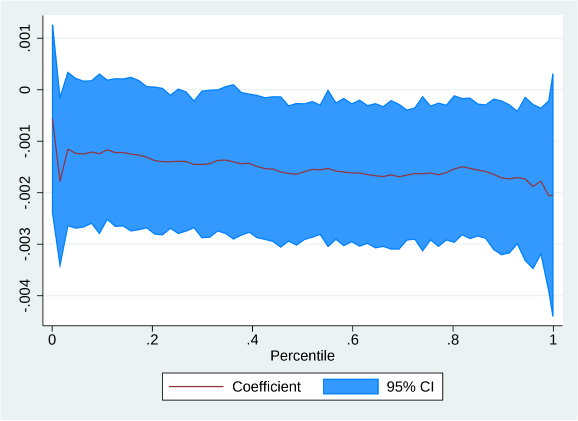

(F) Local Mean Effect of IMR on Consumption

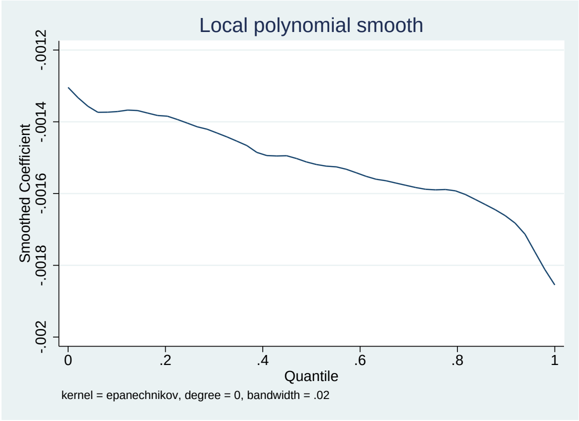

Notes: Panels A through C present quantile regressions with district and year fixed effects, state × urban fixed effects, state time trends, and social group × urban indicators, using the Canay (2011) procedure. Panels D through F present β_i as a function of the wage or consumption distribution, averaged using a smoothed local mean.

Table 7: States Covered by Analysis

State	Observations	Population (millions)	% of Indian Population
Uttar Pradesh	1032	199.8	16.5%
Maharashtra	1131	112.4	9.3%
Bihar	419	104.1	8.6%
West Bengal	861	91.3	7.5%
Andhra Pradesh	1024	84.6	7.0%
Madhya Pradesh	1348	72.6	6.0%
Tamil Nadu	466	72.1	6.0%
Rajasthan	1002	68.5	5.7%
Karnataka	1554	61.1	5.0%
Gujarat	831	60.4	5.0%
Orissa	407	42.0	3.5%
Kerala	553	33.4	2.8%
Jharkhand	341	33.0	2.7%
Punjab	479	27.7	2.3%
Chhatisgarh	546	25.5	2.1%
Haryana	634	25.4	2.1%
Uttarakhand	155	10.1	0.8%

Note: Populations and shares of the total Indian population are as reported in the 2011 Census of India.
